# Phosphoric Metabolites Link Phosphate Import and Polysaccharide Biosynthesis for Candida albicans Cell Wall Maintenance

**DOI:** 10.1128/mBio.03225-19

**Published:** 2020-03-17

**Authors:** Ning-Ning Liu, Maikel Acosta-Zaldívar, Wanjun Qi, Joann Diray-Arce, Louise A. Walker, Theodore J. Kottom, Rachel Kelly, Min Yuan, John M. Asara, Jessica Ann Lasky-Su, Ofer Levy, Andrew H. Limper, Neil A. R. Gow, Julia R. Köhler

**Affiliations:** a Division of Infectious Diseases, Boston Children’s Hospital/Harvard Medical School, Boston, Massachusetts, USA; b Precision Vaccines Program, Boston Children’s Hospital, Boston, Massachusetts, USA; c Aberdeen Fungal Group, Institute of Medical Sciences, Medical Research Council Centre for Medical Mycology at the University of Aberdeen, Aberdeen, United Kingdom; d Thoracic Diseases Research Unit, Departments of Medicine and Biochemistry, Mayo Clinic College of Medicine, Rochester, Minnesota, USA; e Channing Division of Network Medicine, Brigham and Women’s Hospital/Harvard Medical School, Boston, Massachusetts, USA; f Division of Signal Transduction, Beth Israel Deaconess Medical Center, Boston, Massachusetts, USA; g Department of Medicine, Harvard Medical School, Boston, Massachusetts, USA; h Medical Research Council Centre for Medical Mycology at the University of Aberdeen, Institute of Medical Sciences, Aberdeen, United Kingdom; i Mass Spectrometry Core, Beth Israel Deaconess Medical Center, Boston, Massachusetts, USA; University Medical Center Göttingen; Tel Aviv University

**Keywords:** *Candida albicans*, Pho84, antifungal agents, cell wall, chitin synthase, glucan synthase, nucleotide sugar, phosphate metabolism

## Abstract

The Candida albicans high-affinity phosphate transporter Pho84 is required for normal Target of Rapamycin (TOR) signaling, oxidative stress resistance, and virulence of this fungal pathogen. It also contributes to C. albicans’ tolerance of two antifungal drug classes, polyenes and echinocandins. Echinocandins inhibit biosynthesis of a major cell wall component, beta-1,3-glucan. Cells lacking Pho84 were hypersensitive to other forms of cell wall stress beyond echinocandin exposure, while their cell wall integrity signaling response was weak. Metabolomics experiments showed that levels of phosphoric intermediates, including nucleotides like ATP and nucleotide sugars, were low in *pho84* mutant compared to wild-type cells recovering from phosphate starvation. Nonphosphoric precursors like nucleobases and nucleosides were elevated. Outer cell wall phosphomannan biosynthesis requires a nucleotide sugar, GDP-mannose. The nucleotide sugar UDP-glucose is the substrate of enzymes that synthesize two major structural cell wall polysaccharides, beta-1,3- and beta-1,6-glucan. Another nucleotide sugar, UDP-*N*-acetylglucosamine, is the substrate of chitin synthases which produce a stabilizing component of the intercellular septum and of lateral cell walls. Lack of Pho84 activity, and phosphate starvation, potentiated pharmacological or genetic perturbation of these enzymes. We posit that low substrate concentrations of beta-d-glucan- and chitin synthases, together with pharmacologic inhibition of their activity, diminish enzymatic reaction rates as well as the yield of their cell wall-stabilizing products. Phosphate import is not conserved between fungal and human cells, and humans do not synthesize beta-d-glucans or chitin. Hence, inhibiting these processes simultaneously could yield potent antifungal effects with low toxicity to humans.

## Abstract (precis)

*Candida* species cause hundreds of thousands of invasive infections with high mortality each year. Developing novel antifungal agents is challenging due to the many similarities between fungal and human cells. Maintaining phosphate balance is essential for all organisms but is achieved completely differently by fungi and humans. A protein that imports phosphate into fungal cells, Pho84, is not present in humans and is required for normal cell wall stress resistance and cell wall integrity signaling in C. albicans. Nucleotide sugars, which are phosphate-containing building block molecules for construction of the cell wall, are diminished in cells lacking Pho84. Cell wall-constructing enzymes may be slowed by lack of these building blocks, in addition to being inhibited by drugs. Combined targeting of Pho84 and cell wall-constructing enzymes may provide a strategy for antifungal therapy by which two sequential steps of cell wall maintenance are blocked for greater potency.

## INTRODUCTION

*Candida* species are the most commonly isolated invasive human fungal pathogens. Only 3 drug classes are currently available to treat invasive candidiasis, whose attributable mortality is estimated at 19 to 24% (1). Among them, echinocandins, inhibitors of the enzyme beta-1,3-glucan synthase that produces a major cell wall component of *Candida* species, are now recommended as first-line therapy, since they are candidacidal and have few adverse effects or drug interactions (2, 3). Still, outcomes of invasive candidiasis are often poor (2). In fact, early biochemical studies showed that enzymatic activity of beta-1,3-glucan synthase is inhibited by no more than 80% by the echinocandins (4), i.e., echinocandins do not completely inhibit their target (5). Potentiating their effect could be one strategy to improve outcomes in this fearsome infection.

A major barrier to development of new antifungal drugs is the high degree of conservation of many potential drug targets between fungi and humans. We recently found that genetic or pharmacologic interference with the activity of a Candida albicans cell surface phosphate (P_i_) transporter, Pho84, which has no human homolog, can indirectly inhibit TOR complex 1 (TORC1) and thereby selectively target fungal proliferation (7). Loss of Pho84 activity also sensitizes C. albicans to oxidative stress and potentiates the activity of two antifungal agents, the polyene amphotericin B and the echinocandin micafungin (7, 8).

Micafungin inhibits C. albicans beta-1,3-glucan synthase (9). Questioning how Pho84 activity is related to this enzyme, we found that cells lacking *PHO84* poorly tolerated each cell wall stress that we examined. Responsiveness of their cell wall integrity (CWI) pathway signaling through Mkc1 was reduced compared to the congenic wild type. Unlike their oxidative stress hypersensitivity phenotypes (8), homozygous null mutants in *PHO84* (*pho84*^−/−^) did not recover cell wall stress resistance by overexpression of the TORC1-activating GTPase, Gtr1. Hence, cell wall stress hypersensitivity of cells lacking Pho84 was mechanistically distinct from their susceptibility to oxidative stress.

Metabolomics experiments showed that cells lacking Pho84 contained significantly fewer nucleotides and nucleotide sugars than wild-type cells during recovery from P_i_ starvation. Two nucleotide sugars, UDP-glucose and UDP-*N*-acetylglucosamine (UDP-GlcNAc), whose levels in *pho84*^−/−^ cells were decreased, are substrates of the enzymes that produce major cell wall polysaccharides. UDP-GlcNAc is the substrate for chitin synthases (6, 10–14). UDP-glucose is the substrate for beta-1,3-glucan synthases Fks1 and Fks2 (5), as well as for beta-1,6-glucan synthases Kre6 and Skn1 (15–18). We hypothesized that provision of P_i_ contributes to the availability of precursors for beta-d-glucan and chitin biosynthesis.

Our hypothesis predicts that cells lacking P_i_ or Pho84 activity are also hypersensitive to chemical or genetic perturbation of chitin synthase and beta-1,6-glucan synthase, in addition to beta-1,3-glucan synthase inhibition. We focused on the former two biosynthetic processes, since they may be amenable in the future to pharmacologic inhibition, while excellent beta-1,3-glucan synthase inhibitors are already clinically available or in development (19). Lack of Pho84, P_i_ starvation, genetic depletion of these enzymes, and pharmacologic inhibition of chitin synthase had combinatorial effects on C. albicans growth. Pharmacologic inhibition of Pho84 therefore might potentiate not only echinocandin antifungal activity but also that of other inhibitors of cell wall biosynthetic enzymes.

## RESULTS

### A Pho84 contribution to cell wall stress resistance was independent of TORC1.

**FIG 1:**
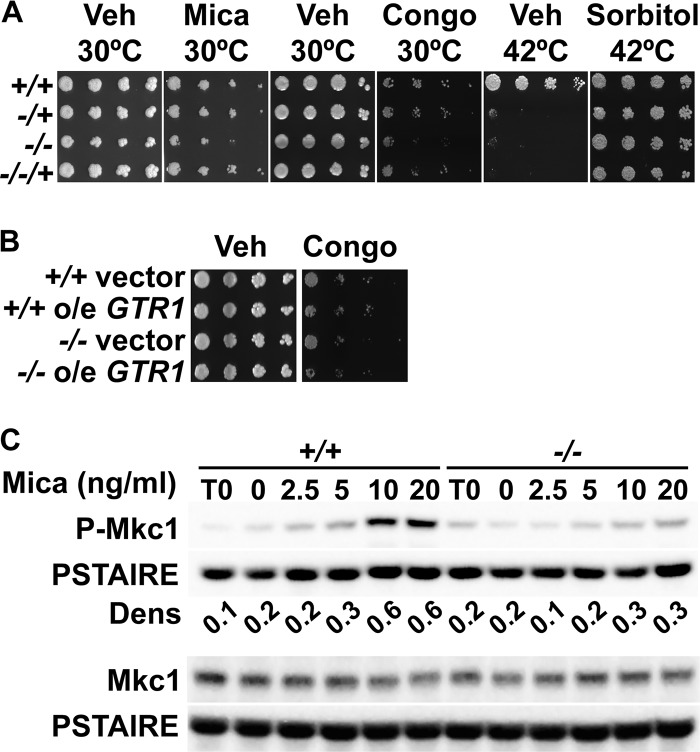
The role of Pho84 in cell wall stress resistance was not mediated by TORC1 but corresponded to weak cell wall integrity signaling. (A) Cell dilutions, shown horizontally, were spotted onto synthetic complete medium (SC) with 1 mM KH_2_PO_4_ with vehicle (Veh) and 25 ng/ml micafungin (Mica), yeast extract-peptone-dextrose (YPD) without or with 50 μg/ml Congo red (Congo), and YPD without or with 1 mM sorbitol and incubated at 30°C (micafungin and Congo red assay) or 42°C (heat resistance and osmotic rescue testing). Strains: +/+, *PHO84/PHO84*, JKC915; −/+, *pho84/PHO84*, JKC1583; −/−, *pho84/pho84*, JKC1450; −/−/+, *pho84/pho84*::*PHO84*, JKC1588. (B) Wild-type (+/+) and *pho84* null mutant (−/−) cells with integrated empty overexpression vector (vector) or overexpressing (o/e) *GTR1* were spotted onto YPD medium with vehicle or 50 μg/ml Congo red (+/+ vector, JKC1594; +/+ o/e *GTR1*, JKC1596; −/− vector, JKC1598; −/− o/e *GTR1*, JKC1600). (C) Western blot analysis of wild-type (+/+, JKC915) and *pho84* null (−/−, JKC1450) cells grown for 90 min in SC with 0.2 mM KH_2_PO_4_ with increasing concentrations of micafungin, probed with antibody to phosphorylated Mkc1 (P-Mkc1), total Mkc1 (Mkc1), and the PSTAIRE antigen of Cdc28 as loading control. Dens, ratio of densitometry of P-Mkc1 to PSTAIRE signal intensity.

C. albicans *PHO84* mRNA levels are upregulated during the interaction with host cells as found by others in *ex vivo* models (20, 21) and *in vivo* during experimental infections (22, 23). Cells lacking Pho84 are attenuated in virulence (8). We tested the responses of mutants in *PHO84* to stresses they might encounter during infection. Having found that loss of Pho84 potentiates the activity of the echinocandin micafungin (7), we asked whether Pho84 has a role in tolerance of other cell wall stressors which do not act through beta-1,3-glucan synthase inhibition. Congo red is an anionic dye which is thought to disrupt fungal cell wall assembly by binding to the cell wall polysaccharide chitin and disrupting the enzymatic reactions that connect chitin to the glucans, thereby weakening the cell wall (24). Cells lacking Pho84 poorly tolerated chemical cell wall stress induced by Congo red exposure (24) and physical stress induced by heat exposure with osmotic rescue (25, 26) (Fig. 1A). These findings indicate that *pho84*^−/−^ mutants are hypersensitive to cell wall stressors that act by diverse mechanisms.

We previously found an activating activity of Pho84 toward TORC1 to be required for rapamycin tolerance (7). Rapamycin hypersensitivity and Sod3 depletion phenotypes of *pho84^−/−^* cells can be suppressed by overexpression of *GTR1*, which encodes a GTPase component of the TORC1-activating EGO complex (7). *GTR1* overexpression had no effect on cell wall stress hypersensitivity of *pho84^−/−^* cells (Fig. 1B), suggesting that Pho84 is required for cell wall stress resistance independently of its TORC1-activating role.

### Pho84 was required for cell wall integrity signaling.

C. albicans cells experiencing cell wall stress induce the CWI signaling pathway (27), whose activity corresponds to the phosphorylation state of its mitogen-activated protein (MAP) kinase, Mkc1 (28). Mkc1 phosphorylation in response to micafungin was weak in cells lacking Pho84 in P_i_-poor medium (Fig. 1C), indicating defective activation of CWI signaling. Decreased Mkc1 phosphorylation was less pronounced but still detectable in rich complex medium yeast extract-peptone-dextrose (YPD), which contains 2 mM inorganic phosphate (P_i_) as well as accessible organic phosphate compounds (29) (see Fig. S1 in the supplemental material), suggesting that lack of Pho84 impacts CWI signaling even in environments of higher P_i_ abundance. C. albicans TORC1 inhibition is known to upregulate Mkc1 phosphorylation (30), so increased sensitivity of *pho84^−/−^* mutants to cell wall stress and their decreased CWI signaling are apparently due to a TORC1-independent mechanism.

10.1128/mBio.03225-19.2 FIG S1 Loss of Pho84 resulted in decreased Mkc1 phosphorylation during micafungin exposure in rich complex medium YPD. Western blot of wild-type (+/+, JKC915) and *pho84* null (−/−, JKC1450) cells. Cells were pregrown in YPD medium overnight and then inoculated into fresh YPD with indicated concentrations of micafungin and grown for 90 min. They were probed with antibody to phosphorylated Mkc1 (P-Mkc1), total Mkc1 (Mkc1), and Cdc28 (PSTAIRE) as loading control. Dens, ratio of densitometry of P-Mkc1 to PSTAIRE signal intensity. Download FIG S1, PDF file, 0.7 MB. Copyright © 2020 Liu et al. 2020 Liu et al. This content is distributed under the terms of the Creative Commons Attribution 4.0 International license.

### Cell walls of *pho84^−/−^* mutants showed decreased alcian blue staining, consistent with decreased cell wall phosphomannan.

**FIG 2:**
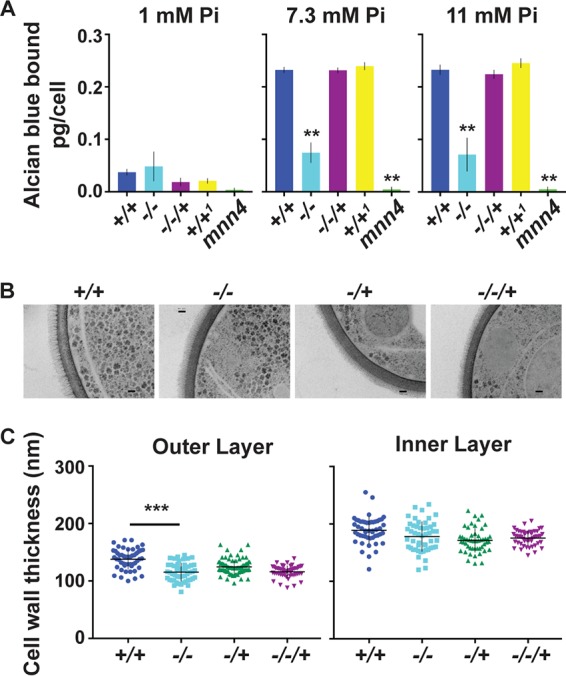
Pho84 was required for a normal phosphomannan cell wall layer. (A) Alcian blue staining. Cells were grown in SC with 1 mM, 7.3 mM, or 11 mM P_i_ for 15 h, and alcian blue staining was assayed in 3 technical replicates. Strains: +/+, *PHO84/PHO84*, JKC915; −/−, *pho84/pho84*, JKC1450; −/−/+, *pho84/pho84*::*PHO84*, JKC1588; +/+^1^, *PHO84/PHO84* CAI-4/CIp10; mnn4, *mnn4*::*hisG-URA3-hisG/mnn4*::*hisG*, CDH5. Representative of 3 biological replicates. (B) Transmission electron micrographs of wild-type (+/+, JKC915), *pho84* null mutant (−/−, JKC1450), *pho84* heterozygote (−/+, JKC1583), or *PHO84* reintegrant (−/−/+, JKC1588) cells. Bar, 100 nm. (C) Thickness measurements of outer and inner cell wall layer of cells (*n* = 50) imaged as in panel B. Error bars show standard deviations (SD).

A major component of C. albicans cell walls is phosphomannan. Phosphodiesters link oligomannosides to glycosylated proteins on the cell surface (31), forming a fibrillar outer layer exterior to the strong chitin and glucan mesh that forms the structural inner layer of the cell wall (32). The phosphomannan component of the C. albicans cell wall confers a negative charge, which can be quantified by binding of the cationic dye alcian blue (33, 34). We questioned whether cells confronting a limiting P_i_ supply might prioritize its use for essential metabolic processes over cell wall construction. Using control cells deleted for *MNN4*, which lack phosphomannan (34), we found decreased alcian blue staining of *pho84^−/−^* cell walls during growth at replete (7.3 mM, the concentration of standard synthetic complete medium [SC]) and excess (11 mM) P_i_ concentrations. At moderate P_i_ concentrations (1 mM), the wild type also produced less cell wall phosphomannan, and the difference between *pho84^−/−^* and wild-type cells was within the sensitivity range of the assay (Fig. 2A). In transmission electron microscopic (TEM) images, the outer cell wall layer of *pho84^−/−^* as well as *pho84^−/+^* and *pho84^−/−/+^* mutant cells was thinner than that of wild-type cells (Fig. 2B and C), suggesting that these cells’ diminished phosphomannan measurably perturbs the cell wall architecture.

### Pho84 was required for production of cell wall polymer precursors.

Mannosylation of proteins and addition of phosphomannan occur in the Golgi apparatus where GDP-mannose is the sugar donor (31). The importance of GDP-mannose availability is evinced by the finding that transport of GDP-mannose into the Golgi lumen is the rate-limiting step in cell surface protein mannosylation (31). More generally, nucleotide sugars are precursors of cell wall polymers, e.g., UDP-glucose for beta-1,6- and beta-1,3-glucan (5, 16) and UDP-GlcNAc for chitin (35). To examine whether loss of Pho84 affects the availability of biosynthetic intermediates required for cell wall production, we compared metabolomes of wild-type and *pho84^−/−^* cells recovering for 4 h in synthetic complete medium (SC) with low (0.22 mM) or excess (11 mM) KH_2_PO_4_ from P_i_ starvation, induced by 3 days’ incubation in SC medium without P_i_ (SC-P_i_) as in the work of Popova et al. (36). This pregrowth period was important in order to unmask P_i_ starvation effects that are otherwise buffered by vacuolar polyphosphate stores in Saccharomyces cerevisiae (37) and are predicted to act similarly in C. albicans (38). Cells grown in this manner, washed in sterile water three times, were extracted three times in 80% methanol for 40 min at −80°C; supernatants were pooled and dried in a SpeedVac and then stored at −80°C until analysis.

**FIG 3:**
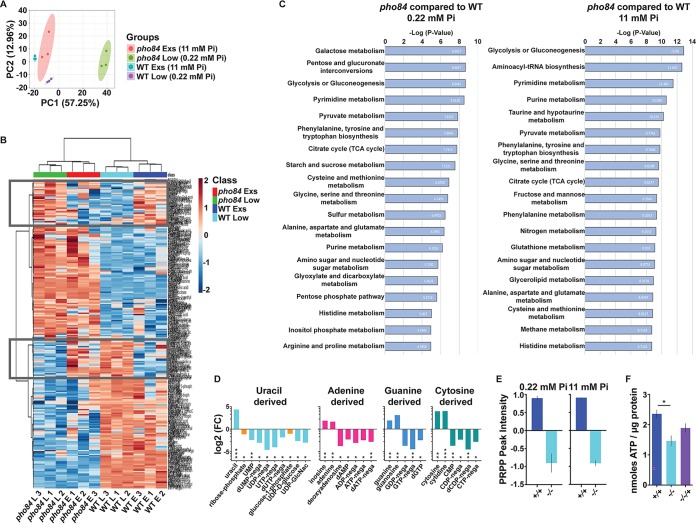
LC-MS/MS measurements of metabolites showed clustering of wild-type versus *pho84* null mutant cells and reflected perturbation of metabolic pathways that contain phosphorylation steps. Cells of the wild type (+/+, JKC915) or *pho84* null mutant (−/−, JKC1450) were grown in SC-P_i_ for 3 days and then fed for 4 h with low (0.22 mM) or excess (11 mM) KH_2_PO_4_ in SC. Cell extracts were subjected to LC-MS/MS for untargeted global metabolomics. (A) Multivariate principal-component analysis shows clustering of profiles according to sample grouping. (B) Unsupervised hierarchical clustering using Euclidean distance of 3 biological replicates for each sample condition (wild type or *pho84* null mutant, low or excess P_i_); red-to-blue scale represents high to low metabolite levels. (C) Summary plot of metabolite set enrichment analysis; significant metabolite groups are ranked according to negative log *P* value. TCA, tricarboxylic acid. (D) Metabolite abundance within biosynthetic pathways of nucleotides and nucleotide sugars, shown as log_2_ of fractional relative intensities of each metabolite in *pho84* null mutant cells versus wild-type cells. ***, *P* (adjusted) < 0.0001; **, *P* (adjusted) = 0.0001 to 0.001; *, *P* (adjusted) = 0.001 to 0.05. (E) Relative abundance of phosphoribosyl-pyrophosphate (PRPP) in *pho84* null mutant (−/−, JKC1450) versus wild-type (+/+, JKC915) cells grown in the stated concentrations of KH_2_PO_4_, shown as scaled, normalized LC-MS/MS peak intensity. (F) ATP concentrations of wild-type (+/+, JKC915), *pho84* null mutant (−/−, JKC1450), or reintegrant (−/−/+, JKC1588) cells grown in rich medium (YPD) for 15 h, expressed as nmol ATP/μg protein. *, *P* = 0.002. Representative of 3 biological replicates.

Hydrophilic interaction liquid chromatography-mass spectrometry (LC-MS/MS) was performed as described in reference 39 to quantitate 258 known metabolites, comparing *pho84^−/−^* cells with wild type. We identified significantly altered compounds and pathways using MetaboAnalyst (40). Principal-component analysis showed clustering of genotypes and of ambient P_i_ availability (Fig. 3A). High reproducibility between experiments and significant differences between wild-type and *pho84^−/−^* cells were observed by arraying the metabolites measured in three biological replicates (from cells grown on different days) in a heat map (Fig. 3B). The heat map illustrates data values scaled by metabolite abundance in arbitrary units across treatments for each metabolite feature. Similarity measure is based on Euclidean distance and clustered using Ward’s linkage.

The genotypes, *pho84^−/−^* mutant and wild type, clustered together primarily (Fig. 3B). Low and excess P_i_ concentrations between the two genotypes did not cluster. Closer inspection showed that within some clusters of metabolites, highlighted by rectangles in Fig. 3B, the relative intensities of metabolites were more similar among *pho84^−/−^* cells in low P_i_ and wild type in excess P_i_ and vice versa than among similar ambient P_i_ concentrations between the two genotypes. Possibly, some metabolic alterations in cells without Pho84 activity may not be due just to lack of P_i_ but also to aberrant regulatory responses.

Biological process enrichment analysis revealed pyrimidine biosynthesis as among the most significantly affected metabolic pathways during growth of *pho84^−/−^* mutant cells at both low and excess ambient P_i_ (Fig. 3C). Purine biosynthesis was also highly significantly altered, as was nucleotide sugar metabolism. We concluded that loss of Pho84 disturbs metabolism of compounds required in cell wall polymer biosynthesis.

Among individual metabolites, purine and pyrimidine nucleotide levels were decreased in *pho84^−/−^* cells compared with wild type, while the bases uracil and cytosine and the nucleosides cytidine and guanosine were substantially increased in *pho84^−/−^* cells (see Table S1 in the supplemental material). For nucleotide products derived from each nucleobase, we observed accumulation of metabolites before a phosphorylation step and their depletion after this step (Fig. 3D). The most important phosphoric nucleotide precursor, phosphoribosyl-pyrophosphate (PRPP), was sharply decreased (Fig. 3E). These results were obtained using MetaboAnalyst; the column-wise means of all samples from *pho84* null mutant cells were divided by the column-wise means of all samples from wild-type cells before column normalization; absolute value changes were compared as fold change. The constellation of metabolic intermediates that we observed suggested that lack of nucleotides was due to a dearth of P_i_, since intermediates destined for phosphorylation seemed to have accumulated upstream of the cognate kinase. Degradation products of purines like allantoin were strongly decreased, suggesting that purine salvage was highly upregulated (Table S1).

10.1128/mBio.03225-19.8 TABLE S1 Metabolites of significantly different abundance. Using MetaboAnalyst, the column-wise means of all samples from *pho84* null mutant cells were divided by the column-wise means of all samples from wild-type cells before column normalization; absolute value changes were compared as fold change. Metabolites with a significant difference between *pho84* null mutant and wild-type cells are listed. Download Table S1, PDF file, 0.1 MB. Copyright © 2020 Liu et al. 2020 Liu et al. This content is distributed under the terms of the Creative Commons Attribution 4.0 International license.

To test the apparent lack of nucleotides independently, we measured the concentration of the most important nucleotide, ATP, in cells grown to saturation in rich complex medium as in the work of Grahl et al. (41). *pho84^−/−^* cells contained substantially less ATP than wild-type or −/−/+ *PHO84* reintegrant cells (Fig. 3F), confirming the result of the LC-MS/MS experiments. Metabolic derangements of *pho84^−/−^* cells were extensive and involved multiple further biosynthetic pathways (Fig. 3C and Table S1). Since ATP participates in a majority of metabolic processes (42), its decreased availability could drive many of the metabolic effects that we observed.

### Loss of Pho84 decreased the amount of detectable cell wall chitin.

UDP-GlcNAc, the substrate of chitin synthases, was the most significantly decreased cell wall precursor requiring a pyrimidine in *pho84^−/−^* cells (Table S1). We hypothesized that if P_i_ to produce specific pyrimidine nucleotide sugars is insufficient, the cell wall polymers produced from these nucleotide sugars will be diminished. To measure cells’ chitin content, we modified an assay described in reference 43, using wild-type cells with decreased chitin content for validation. Cells were grown overnight in SC with 0.5 mM P_i_, unexposed or exposed to increasing concentrations of the chitin synthase inhibitor nikkomycin Z (nikkomycin) (Fig. S2A) (44, 45), a competitive inhibitor of C. albicans chitin synthases (44). Fluorescence intensity of calcofluor white-stained cells, recorded by flow cytometry, clearly reflected the decreased chitin content and dose response of nikkomycin-exposed cells (Fig. S2A).

**FIG 4:**
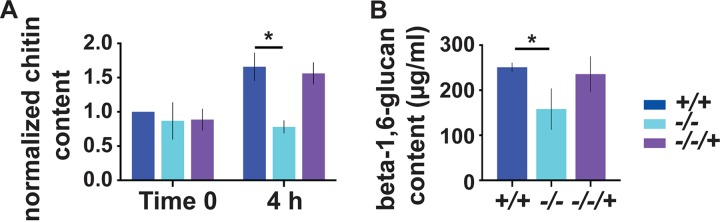
Pho84 activity contributed to cell wall chitin and beta-1,6-glucan synthesis. (A) Chitin content of cells of indicated *PHO84* genotypes. Chitin staining with calcofluor white was quantified by flow cytometry of ≥10^5^ events; fluorescence intensity signals were normalized using the wild-type time zero readings for each of 3 biological replicates whose combined results are shown. (B) Alkali-insoluble beta-1,6-glucan content of cells grown as in panel A was measured by ELISA. Error bars show SD for 3 biological replicates. *, *P* < 0.05. +/+, wild type, JKC915; −/−, *pho84* null mutant, JKC1450; −/−/+, *PHO84* reintegrant, JKC1588.

10.1128/mBio.03225-19.3 FIG S2 An assay to quantify cell wall chitin content by flow cytometry following calcofluor white staining. (A) Effect of nikkomycin on calcofluor white fluorescence of wild-type cells. Wild-type cells (JKC915) were inoculated to a final OD_600_ of 0.2 in SC with moderate (0.5 mM) P_i_ with indicated concentrations of nikkomycin and grown overnight. Fixed cells were stained with calcofluor white. Chitin staining was measured using flow cytometry, and the signals were normalized using the vehicle (Veh, 0 nikkomycin) readings for each biological replicate. Error bars show standard deviations for 3 biological replicates. *, *P* < 0.05. (B) Flow cytometry histograms of calcofluor white-stained cells in Fig. 4A. Representative of 3 biological replicates. *+/+*, *PHO84/PHO84*, JKC915; −/−, *pho84/pho84*, JKC1450; −/−/+, *pho84/pho84*::*PHO84*, JKC1588. Download FIG S2, PDF file, 0.3 MB. Copyright © 2020 Liu et al. 2020 Liu et al. This content is distributed under the terms of the Creative Commons Attribution 4.0 International license.

We then measured the chitin content in wild type, *pho84^−/−^* mutant, and −/−/+ *PHO84* reintegrant cells recovering from P_i_ starvation, as in the metabolomics experiments, during growth in SC with low (0.22 mM) P_i_ for 4 h. Cells without Pho84 had a significantly lower chitin cell wall content than wild-type and reintegrant cells (Fig. 4A and Fig. S2B). Chitin quantitation results were highly numerically reproducible among biological replicates performed on different days (Fig. 4A), when measurements were normalized to the mean fluorescence of wild-type cells harvested at the end of P_i_ starvation (time zero). This result suggests a robust metabolic or signaling-based regulatory system that directs biosynthetic fluxes in P_i_-starved *pho84^−/−^* cells away from chitin production and provides a possible causal link to the role of Pho84 in cell wall stress resistance.

### Less beta-1,6-glucan was detected in *pho84^−/−^* cell walls.

In Saccharomyces cerevisiae, abundant covalent linkages of beta-1,6-glucan to 3 other cell wall components, beta-1,3-glucan, chitin, and mannoproteins, suggested a critical structural function of beta-1,6-glucan as the central “glue” for the distinct polymers that make up the cell wall (46). Beta-1,6-glucan, comprising >50% of alkali-insoluble cell wall glucan in C. albicans (16), is produced by two conserved homologous synthases, Kre6 and Skn1 (16, 18), whose S. cerevisiae homologs utilize UDP-glucose as the substrate (15, 47). An enzyme-linked immunosorbent assay (ELISA) used in Pneumocystis carinii (48) was adapted to compare the beta-1,6-glucan contents of wild-type and *pho84^−/−^* cells recovering from P_i_ starvation; a strain in which *KRE6* transcription was repressible from the *MAL2* promoter (*pMAL2*) served as the control in establishing the assay (Fig. S3A).

10.1128/mBio.03225-19.4 FIG S3 Cell wall beta-1,6-glucan content measurement. (A) ELISA of beta-1,6-glucan content in control strains (+/+, wild type JKC915; *kre6/pMAL2-KRE6 skn1*^−/−^, JKC2389). Cells were grown for 20 h in 2× SC-0.2 mM P_i_-2% glucose. Beta-1,6-glucan content of alkaline-soluble and -insoluble fractions was measured by ELISA (upper panel). Error bars show SD for 2 technical replicates. *, *P* < 0.05. The same fractions were also qualitatively analyzed by dot blot (lower panel) using an anti-beta-1,6-glucan antibody. (B) Cells grown as described in Fig. 4 were harvested after 4 h of growth, and the alkaline-soluble fraction of beta-1,6-glucan content was measured by ELISA. Error bars show SD for 3 biological replicates. *, *P* < 0.05. *+/+*, *PHO84/PHO84*, JKC915; −/−, *pho84/pho84*, JKC1450; −/−/+, *pho84/pho84*::*PHO84*, JKC1588. Download FIG S3, PDF file, 0.4 MB. Copyright © 2020 Liu et al. 2020 Liu et al. This content is distributed under the terms of the Creative Commons Attribution 4.0 International license.

Fungal cell wall components are classically analyzed from 2 fractions, alkali insoluble and soluble; the major alkali-insoluble fraction represents a mesh of chitin fibrils covalently linked to glucans (49, 50) which provides the structural stability and shape to the cell wall (51). In S. cerevisiae, the covalent bond lending insolubility, in hot NaOH, to this cell wall fraction consists of chitin linked to the nonreducing end of a beta-1,3-glucan chain (52). We quantified beta-1,6-glucan in alkali-insoluble cell wall fractions. Alkali-insoluble cell wall fractions from *pho84^−/−^* cells contained significantly less beta-1,6-glucan than those from wild-type cells (Fig. 4B). Additionally, we examined alkali-soluble fractions. The alkali-soluble cell wall fraction of C. albicans comprises 5 to 11% of the cell wall mass depending on growth conditions (53) and represents glucans unlinked to the chitin-glucan mesh that forms the mechanoresistant cell wall core. The alkali-soluble cell wall fraction of *pho84^−/−^* mutant cells contained more beta-1,6-glucan than that of the wild type (Fig. S3B), suggesting a reduction in covalent linkages among the major cell wall polysaccharides in these cells; the reduction in cell wall chitin content (Fig. 4A and Fig. S2B) may be responsible for this finding. Overall, major cell wall structural polysaccharides were diminished in cells lacking Pho84 activity, apparently paralleling the availability of their metabolic precursors.

### Phosphate deprivation sensitized wild-type cells to pharmacologic inhibition of beta-1,3-glucan and chitin synthesis.

If *pho84^−/−^* cells are hypersensitive to beta-1,3-glucan synthase inhibition because they lack P_i_ for production of precursors, depriving wild-type cells of P_i_ should have a similar effect. Activity of cell wall polysaccharide-synthetic enzymes decreases when they bind a specific inhibitor (4). By mass action, accumulation of their product should diminish further with declining concentrations of their substrates, i.e., when UDP-glucose and UDP-GlcNAc concentrations drop. Micafungin was used to inhibit beta-1,3-glucan synthase, and nikkomycin was used to inhibit chitin synthase. We had no pharmacological inhibitor of beta-1,6-glucan synthase since the only published such compound (54) is no longer available.

We questioned wild-type cells’ responses under conditions that physiologically diminish the role of Pho84, using conditions where its expression in wild-type cells is low. We first established the highest P_i_ concentration at which C. albicans derepresses *PHO84* transcription, expecting that, as in S. cerevisiae*, PHO84* is repressed in high ambient P_i_ concentrations (37). Using a *PHO84* promoter-green fluorescent protein (GFP) fusion, we determined that the *PHO84* promoter became derepressed at ≤0.4 mM ambient P_i_ (Fig. S4); hence, we used 0.5 mM as a moderate P_i_ concentration during refeeding of P_i_-starved cells. Wild-type cells starved for P_i_ in the same way as for the metabolomics experiments, or prefed with excess (12 mM) P_i_, were reinoculated into moderate (0.5 mM) or excess (12 mM) P_i_ concentrations and exposed to inhibitors of beta-1,3-glucan- and of chitin synthesis. Cells were incubated in 2× SC with 2% glucose in these experiments, in order to optimize nutrients during inhibitor exposure.

10.1128/mBio.03225-19.5 FIG S4 *PHO84* expression and cellular growth were tightly controlled by the ambient P_i_ concentration. Cells expressing GFP under the control of the *PHO84* promoter (JKC1659) were pregrown in YPD with added 10 mM P_i_ overnight before dilution into SC with indicated P_i_ concentrations. The fluorescent signal and OD_600_ were followed over 30 h. Representative of 3 biological replicates. Download FIG S4, PDF file, 0.5 MB. Copyright © 2020 Liu et al. 2020 Liu et al. This content is distributed under the terms of the Creative Commons Attribution 4.0 International license.

**FIG 5:**
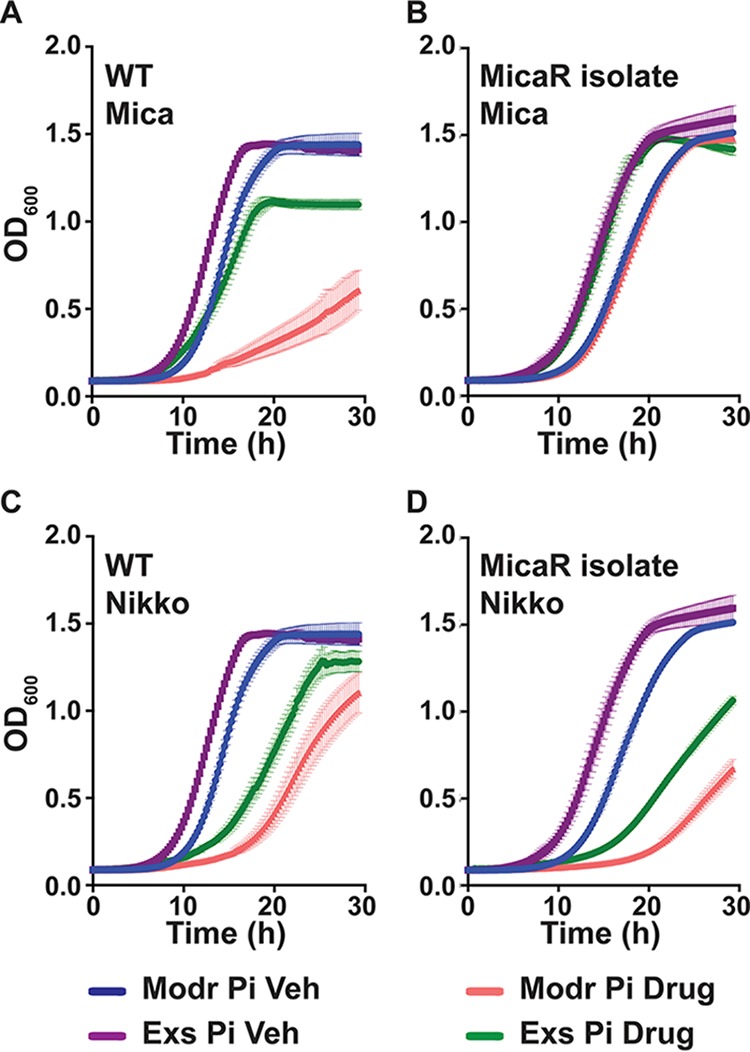
P_i_ starvation sensitized wild-type cells to inhibitors of two cell wall polysaccharide synthetic enzymes. (A and B) Growth of cultures exposed to vehicle or 40 ng/ml micafungin of the wild type (JKC915) or a micafungin-resistant bloodstream isolate (MicaR, JKC2490), pregrown in 2× SC without or with excess P_i_ for 1 day and inoculated into 2× SC with moderate (Modr) or excess (Exs) P_i_, respectively. (C and D) Growth of cells exposed to vehicle or 8 μM nikkomycin of the wild type (JKC915) or a micafungin-resistant bloodstream isolate (JKC2490) treated as in panels A and B. Modr P_i_, pregrown in 0 P_i_, inoculated to 0.5 mM P_i_ with vehicle or drug; Exs P_i_, pregrown with 12 mM P_i_, inoculated to 12 mM P_i_ with vehicle or drug. Representative of 3 biological replicates; error bars show SD for 3 technical replicates.

Wild type cells prestarved for P_i_ before, and refed moderate P_i_ during, exposure to micafungin were significantly more sensitive than control cells provided with excess P_i_ throughout the experiment (Fig. 5A). However, P_i_ starvation during micafungin exposure did not lead to growth defects of a micafungin-resistant C. albicans bloodstream isolate from a patient treated long-term with this drug (Fig. 5B). This finding indicates that P_i_ starvation did not cause global growth defects in these experiments; it potentiated the effect of specific inhibitors of enzymes whose substrates are linked to P_i_ availability.

Growth defects induced by nikkomycin exposure were enhanced in cells prestarved for P_i_ and refed with moderate P_i_ (Fig. 5C), in wild-type cells as well as in the micafungin-resistant bloodstream isolate (Fig. 5D). Hence, P_i_ starvation sensitized wild-type cells to inhibition of two distinct sugar nucleotide-consuming cell wall biosynthetic processes.

### Loss of Pho84 and P_i_ starvation sensitized cells to genetic depletion of chitin and beta-1,6-glucan synthases.

If *pho84^−/−^* cells’ hypersensitivity to cell wall stressors is due to insufficient concentrations of nucleotide sugars, cells with diminished activity of chitin and beta-1,6-glucan synthases should be hypersensitive to loss of Pho84 and to P_i_ starvation. To further test this idea, we perturbed these enzymes genetically.

**FIG 6:**
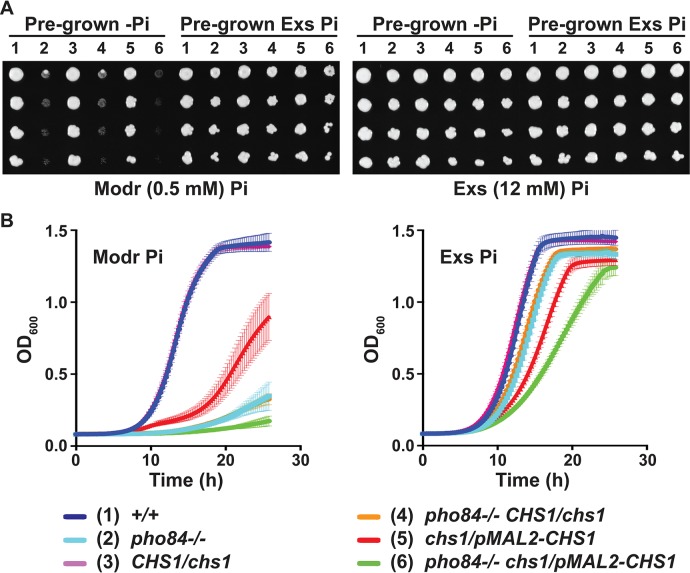
Growth defects of cells depleted of *CHS1* were potentiated by lack of Pho84 activity. (A) Cells of indicated genotypes were pregrown in 2× SC-2% maltose without or with excess P_i_ for 1 day, and dilutions were spotted on 2× SC-2% glucose medium containing moderate or excess P_i_. Dilutions are shown vertically. (B) Cells pregrown without or with excess P_i_ as described in panel A were inoculated into 2× SC-2% glucose containing moderate or excess P_i_, respectively. Representative of 3 biological replicates; error bars show SD for 3 technical replicates. *+/+*, JKC915; *pho84*^−/−^, JKC1450; *chs1/CHS1*, JKC2128; *pho84*^−/−^ *chs1/CHS1*, JKC2141; *chs1/pMAL2-CHS1*, JKC2216; *pho84*^−/−^ *chs1/pMAL2-CHS1*, JKC2235.

Among the 4 chitin synthases of C. albicans, the only essential isoenzyme, Chs1, is required for septum production during cell division and contributes to the stability of lateral cell walls (55). We constructed mutants whose only *CHS1* allele is controlled by *pMAL2* or by *tetO*, repressible by glucose or doxycycline, respectively, and confirmed that they exhibit previously described phenotypes (13, 55) (Fig. S5). The effect of P_i_ availability during *CHS1* depletion was examined. *CHS1* was depleted from *pMAL2* or from *tetO* after a day of P_i_ starvation or P_i_ excess feeding during which *CHS1* expression was induced from these promoters, by incubation in maltose or in the absence of doxycycline. *CHS1*-depleted cells, incubated in glucose or doxycycline, respectively, had a significant growth defect in a moderate P_i_ concentration (0.5 mM) (Fig. 6A and B and Fig. S6). The specificity of the P_i_-dependent growth defect of *CHS1*-depleted cells was demonstrated by comparatively robust growth of these cells fed excess P_i_ (Fig. 6A and B).

10.1128/mBio.03225-19.6 FIG S5 Cells depleted of *CHS1* were defective in filamentous growth, developed ballooning filaments, and lysed during nikkomycin exposure. (A) Filamentation of wild-type cells (+/+, JKC915) and *chs1/pMAL2-CHS1* (JKC2280) on solid RPMI medium (pH 5.5) with 1.8% maltose and 0.2% glucose or 2% glucose for 8 days at 37°C. Bar, 200 μm. (B) Filamentation of wild-type cells (+/+, JKC915) and *chs1/tetO-CHS1* (JKC2272) on solid RPMI medium (pH 5.5) with 2% glucose and vehicle or 1 μg/ml doxycycline (Dox) to repress transcription from *tetO* for 5 days at 37°C. Bar, 200 μm. (C) Colonies of wild-type cells (+/+, JKC915) and *chs1/tetO-CHS1* (JKC2272) on solid YPD or Spider medium with vehicle or 30 μg/ml doxycycline for 1 day of 30°C incubation for YPD and 37°C incubation for Spider medium plates. Bar, 200 μm. (D) Morphologies of wild-type cells (+/+, JKC915) and *chs1/pMAL2-CHS1* (JKC2216) in standard SC with glucose or maltose and vehicle or nikkomycin for 30 h at 37°C. Panels A to D are representative of 3 biological replicates. Download FIG S5, PDF file, 2.0 MB. Copyright © 2020 Liu et al. 2020 Liu et al. This content is distributed under the terms of the Creative Commons Attribution 4.0 International license.

10.1128/mBio.03225-19.7 FIG S6 Growth defects of *CHS1*-depleted cells were potentiated by loss of Pho84 and rescued by excess P_i_ loading. Cells of indicated genotypes were pregrown in 2× SC-2% maltose without or with excess (12 mM) P_i_ for 1 day and inoculated into 2× SC-2% glucose with moderate (0.5 mM) or excess P_i_, respectively, and vehicle or 500 ng/ml doxycycline (Dox). Modr P_i_, pregrown without P_i_, inoculated to moderate P_i_; Exs P_i_, pregrown with excess P_i_, inoculated to excess P_i_. Representative of 3 biological replicates; error bars show SD for 3 technical replicates. *+/+*, JKC915; *pho84*^−/−^, JKC1450; *chs1/tetO-CHS1*, JKC2212; *pho84*^−/−^ *chs1/tetO-CHS1*, JKC2288. Download FIG S6, PDF file, 1.8 MB. Copyright © 2020 Liu et al. 2020 Liu et al. This content is distributed under the terms of the Creative Commons Attribution 4.0 International license.

The role of Pho84 in cells depleted for *CHS1* was probed. Loss of *PHO84* potentiated the growth defects of cells depleted of *CHS1* even in cells fed excess P_i_ (Fig. 6A and B). These experiments suggest that as Chs1 activity became limiting because of a decline of its cognate transcript, P_i_ availability impacted growth significantly. Additionally, a Pho84-specific role seemed to emerge that was independent of ambient P_i_ concentrations.

**FIG 7:**
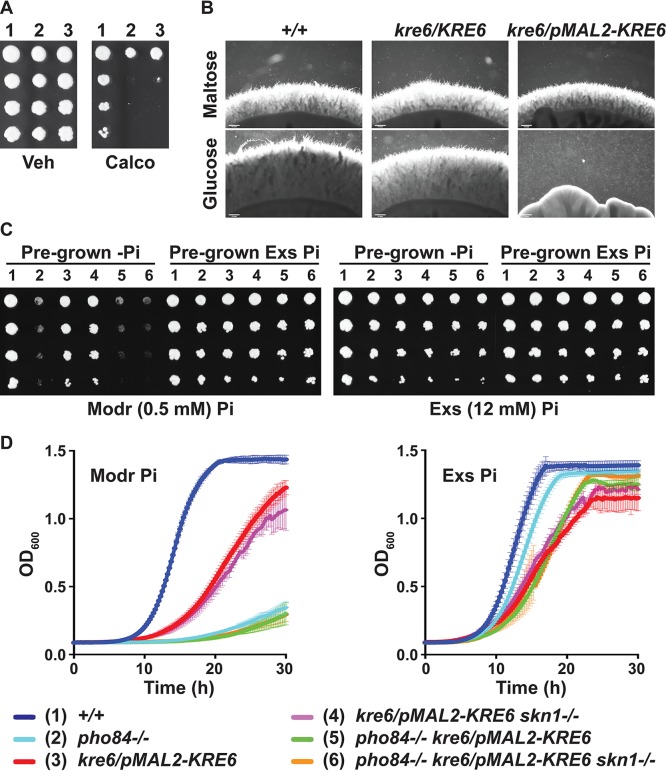
*KRE6* depletion resulted in filamentation defects and in growth defects potentiated by lack of Pho84 activity. (A) Wild-type cells (1, JKC915) and independent heterozygotes *kre6/KRE6* (2, JKC2111; 3, JKC2113) were spotted on YPD medium with vehicle or 30 μg/ml calcofluor white. (B) Filamentation of wild-type (+/+, JKC915), *kre6/KRE6* (JKC2111), and *kre6/pMAL2-KRE6* (JKC2204) cells on RPMI agar medium (pH 5.5) with 1.8% maltose and 0.2% glucose or 2% glucose, for 7 days at 37°C. Bar, 200 μm. (C) Cells of indicated genotypes (see bottom of figure) were pregrown in 2× SC-2% maltose without or with excess (12 mM) P_i_ for 3 days, and dilutions were spotted on 2× SC-2% glucose containing moderate (0.5 mM) or excess P_i_. (D) Cells pregrown without or with excess P_i_ as in panel C were inoculated into 2× SC-2% glucose containing moderate or excess P_i_, respectively. Representative of 3 biological replicates; error bars show SD for 3 technical replicates. *+/+*, JKC915; *pho84*^−/−^, JKC1450; *kre6/pMAL2-KRE6*, JKC2204; *kre6/pMAL2-KRE6 skn1*^−/−^, JKC2389; *pho84*^−/−^ *kre6/pMAL2-KRE6*, JKC2464; *pho84*^−/−^ *kre6/pMAL2-KRE6 skn1*^−/−^, JKC2468.

We constructed strains in which a single allele of the gene encoding the major beta-1,6-glucan synthase, *KRE6* (16, 18), is controlled by *pMAL2*. Additionally, the gene encoding the second known beta-1,6-glucan synthase, *SKN1*, was deleted in the *kre6/pMAL2-KRE6* background (18). We observed more severe growth and filamentation phenotypes than did Han et al. (18) during exposure of 2 independent *kre6/KRE6* heterozygous deletion mutants to calcofluor white (Fig. 7A and B), and during depletion of *KRE6* from *pMAL2* in glucose. To reexamine our findings in light of these discordant results, we constructed strains in which a single *KRE6* allele is transcribed from repressible *tetO*. While neither of these repressible promoters can completely shut off transcriptional activity (56), the phenotypes we observed during repression of *KRE6* transcription from either promoter were inconsistent with those of Han et al.; phenotypes of 2 independently constructed lineages from 2 *kre6/KRE6* heterozygous strains were indistinguishable. In contrast to the findings of Han et al. (18), additional deletion of *SKN1* contributed little to growth defects of *KRE6*-depleted cells under these experimental conditions (Fig. 7C and D).

To examine the effect of P_i_ availability on cells with decreased beta-1,6-glucan synthase activity, cells were P_i_ starved as for the metabolomics experiments but in 2× SC to allow for maximal growth and recovered in moderate (0.5 mM) or excess (12 mM) P_i_. Control cells were P_i_ loaded in 12 mM P_i_. Cells depleted for *KRE6* after P_i_ starvation and during recovery in moderate P_i_ concentrations had more severe growth defects than cells that were continuously fed excess P_i_ (Fig. 7C and D). This result suggested a need for P_i_ when levels of beta-1,6-glucan synthase became limiting, in order to supply sufficient concentrations of the enzyme’s substrate, UDP-glucose.

### Loss of Pho84 prevented compensatory chitin deposition in cells depleted for beta-1,6-glucan.

Inhibiting beta-1,3-glucan synthesis pharmacologically with echinocandins or depleting beta-1,6-glucan synthases genetically induces compensatory synthesis of chitin by both transcriptional and posttranscriptional mechanisms (57–59). We measured chitin content of cells with and without *PHO84*, which were depleted for *KRE6* with or without intact *SKN1* loci (*kre6*/*pMAL2-KRE6* and *kre6*/*pMAL2-KRE6 skn1/skn1*, as well as *pho84/pho84 kre6*/*pMAL2-KRE6* and *pho84/pho84 kre6*/*pMAL2-KRE6 skn1/skn1*). Cells were precultured as for metabolomics experiments, except that maltose was provided as the carbon source to permit expression of *KRE6*, and 2× SC was used to allow for maximal provision of nutrients other than P_i_. They were then grown for 8 h in 2× SC with 0.22 or 11 mM P_i_ as in metabolomics experiments, using glucose as the carbon source to repress *KRE6*.

**FIG 8:**
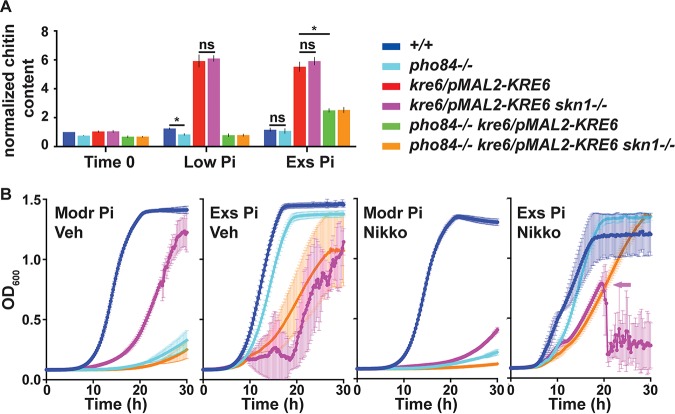
During *KRE6* depletion, Pho84 contributed to compensatory chitin synthesis and growth. (A) Cellular chitin content. After 3 days’ passages in 2× SC-P_i_-2% maltose (time zero), cells were grown for 8 h in 2× SC-2% glucose with low (0.22 mM) or excess (11 mM) P_i_. Fluorescence intensity of calcofluor white-stained cells was measured by flow cytometry and normalized to wild-type time zero readings for each biological replicate. Error bars show SD for 3 biological replicates. *, *P* < 0.05; ns, not significant. (B) Growth during *KRE6* depletion, loss of Pho84, and chitin synthase inhibition. After 3 days’ passages in 2× SC-2% maltose without or with excess P_i_, cells were inoculated into 2× SC-2% glucose with moderate or excess P_i_, respectively, with vehicle (Veh) or 0.25 μM nikkomycin (Nikko). Modr P_i_, pregrown without P_i_, inoculated to moderate (0.5 mM) P_i_; Exs P_i_, pregrown with excess (12 mM) P_i_, inoculated to excess P_i_. Representative of 3 biological replicates; error bars show SD for 3 technical replicates. *+/+*, JKC915; *pho84*^−/−^, JKC1450; *kre6/pMAL2-KRE6*, JKC2204; *kre6/pMAL2-KRE6 skn1*^−/−^, JKC2389; *pho84*^−/−^ *kre6/pMAL2-KRE6*, JKC2464; *pho84*^−/−^ *kre6/pMAL2-KRE6 skn1*^−/−^, JKC2468. Arrow in last panel of panel B indicates onset of overwhelming filamentation, at which time OD_600_ ceased to reflect the cell number.

We found a higher chitin content in cells depleted of *KRE6*, as previously reported (59). This effect was completely or partially abrogated in cells lacking Pho84 (Fig. 8A), depending on the ambient P_i_ concentration. Presence or absence of *SKN1* had no effect (Fig. 8A). We concluded that compensatory chitin synthesis in cells whose beta-1,6-glucan biosynthesis was diminished required the availability of sufficient P_i_, as well as an activity of Pho84.

### Loss of Pho84 and chitin synthase inhibition potentiated growth defects during beta-1,6-glucan synthase depletion.

When beta-1,3-glucan synthase is inhibited by an echinocandin, upregulation of chitin synthesis can compensate for loss of cell wall stability and inhibition of growth (57). We examined the effects of blocking compensatory mechanisms for loss of beta-1,6-glucan. Beta-1,6-glucan synthase was depleted by repressing *KRE6* from *pMAL2*, and Pho84 activity was eliminated genetically, while chitin synthase was inhibited with low concentrations of nikkomycin. Loss of Pho84 activity and chitin synthase inhibition each further reduced growth of cells lacking beta-1,6-glucan synthase (Fig. 8B). We concluded that the inhibitory effects of beta-1,6-glucan synthase depletion are potentiated by inhibition of chitin synthase and loss of Pho84 activity.

## DISCUSSION

Cells lacking Pho84 are hypersensitive to cell wall stress (7) (Fig.1A). Our mechanistic analysis of this phenotype indicated that, unlike their oxidative stress hypersensitivity, it was not directly related to these cells’ diminished TORC1 signaling (Fig. 1B). While oxidative stress signaling is upregulated in *pho84^−/−^* cells (8), their cell wall integrity signaling was abnormally weak as measured by the phosphorylation state of the CWI MAP kinase Mkc1 (Fig. 1C). Dampened CWI signaling in *pho84^−/−^* cells cannot be explained by their decreased TORC1 signaling activity, because TORC1 inhibition induces signaling through Mkc1 (30). Decreased alcian blue staining of *pho84^−/−^* mutants suggested that the phosphomannoprotein content of their cell walls was decreased (Fig. 2A). Significant thinning of their outer phosphomannan cell wall layer was found by measurements of TEM images (Fig. 2B and C). This thinning was far less striking than that seen in TEM micrographs of mutants in enzymes that produce this layer, e.g., in the Mnn2 family of mannosyltransferases (60). Nevertheless, the difference in outer layer thickness between wild-type and *pho84^−/−^* cells was highly significant (Fig. 2C). The phenotype was similar in cells lacking one or both copies of *PHO84*. We noted striking haploinsufficiency of *pho84/PHO84* cells (and of *pho84/pho84*::*PHO84* cells) for outer cell wall layer thickness; haploinsufficiency is known to affect both structural and regulatory genes in C. albicans (61, 62). We concluded that lack of Pho84 can disturb the normal cell wall architecture.

This finding prompted the idea that cells lacking Pho84 are defective in synthesizing cell wall components that require phosphorylated precursors. Structural cell wall polysaccharides of C. albicans, beta-1,6-glucan, beta-1,3-glucan, and chitin in order of their abundance (16), are synthesized from monosaccharide precursors activated with UTP to generate UDP-containing nucleotide sugars; UTP biosynthesis requires availability of the P_i_ donor ATP. Vacuolar polyphosphate stores buffer decreased extracellular P_i_ availability in S. cerevisiae (37); vacuolar polyphosphate storage pools are also present in C. albicans (38). Hence, we applied the protocol of Popova et al. (36) to neutralize intracellular P_i_ stores before an incubation period in low, moderate, or excess P_i_. Metabolomics experiments showed derangements of biosynthetic pathways that require phosphorylation steps in *pho84^−/−^* cells (Fig. 3). Pyrimidine biosynthesis was highly significantly altered. The sugar nucleotides that act as precursors for cell wall polysaccharide biosynthesis, UDP-glucose and UDP-GlcNAc, were decreased 2.6- and 2.9-fold, respectively, in *pho84^−/−^* cells in our experiments (Table S1). Our findings agree with those of Boer et al., who found decreased levels of nucleotides and of the nucleotide sugar UDP-glucose in P_i_-limited S. cerevisiae cells grown in continuous culture in a chemostat (63).

We considered whether accumulation of toxic metabolites might account for cell wall biosynthesis defects. However, toxic metabolites that accumulate, e.g., in S. cerevisiae models of galactosemia (64, 65) and fructose intolerance (65), are sugar phosphates, i.e., their biosynthesis requires a phosphorylation step whose substrate was scarce in cells lacking Pho84. Accordingly, we did not identify potentially toxic metabolites among the significantly dysregulated metabolites in these cells (Table S1).

Perturbation of multiple other metabolic processes was observed. This result is consistent with depletion of ATP, a major P_i_ donor and energy currency of the cell, which we confirmed in independent assays for *pho84^−/−^* cells (Fig. 3F). Glycolysis and galactose and pentose processing were disrupted (Fig. 3C), consistent with a requirement for phosphorylation in metabolism of these sugars; glycolysis alone consumes 2 P_i_ and 2 ADP molecules per molecule of glucose (66). Possibly, defects in producing sugar precursors of nucleotide sugars may contribute to the disruption of nucleotide biosynthesis that we observed in cells lacking Pho84. Insufficient ATP to produce nucleotide sugars may also be ultimately responsible for cell wall phenotypes of cells perturbed in mitochondrial function, as reported, e.g., in references [Bibr B67][Bibr B68][Bibr B72]. However, ATP deprivation is expected to impact *pho84^−/−^* cells’ fitness in multiple processes beyond diminished cell wall precursor availability; its effect in C. albicans’ different natural niches and life cycle stages remains to be explored in future experiments.

**FIG 9:**
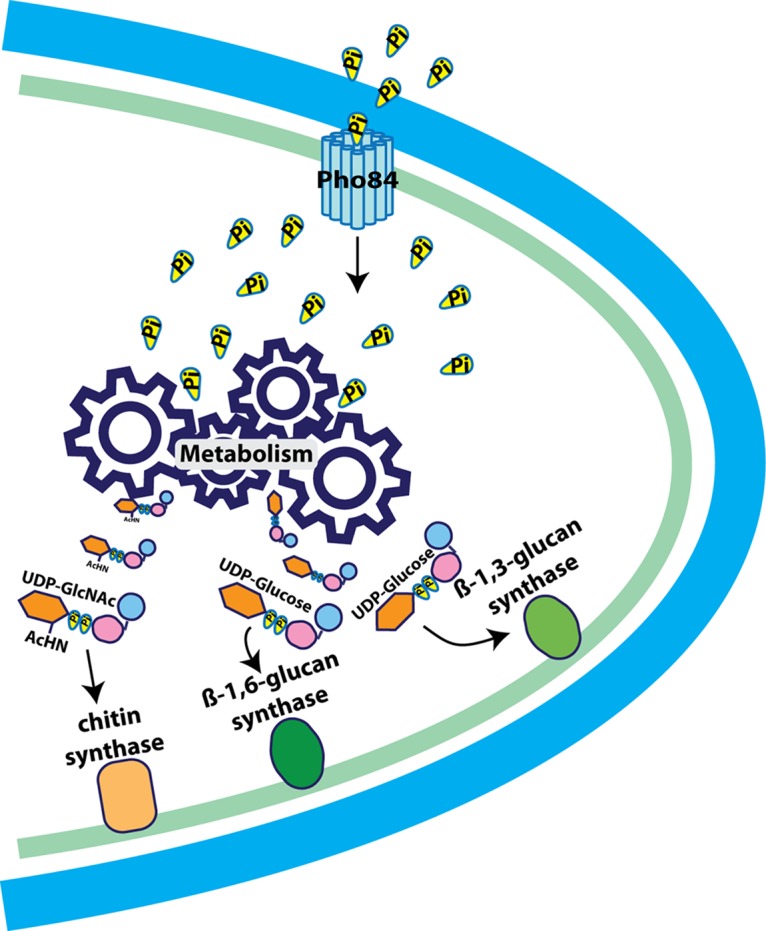
Model: role of Pho84 in cell wall polysaccharide biosynthesis. P_i_ is imported across the cytoplasmic membrane by Pho84, where it is available to metabolic processes that produce the nucleotide sugars UDP-glucose and UDP-GlcNAc. These nucleotide sugars are the substrates of glucan and chitin synthases that produce major polysaccharides of the cell wall, thereby providing its structural stability.

The metabolomics findings suggested a simple model, by which lack of monomeric precursors for cell wall polysaccharides deprives the cognate enzymes of their substrates, slowing the reaction velocity and leading to decreased structural cell wall polysaccharides and hence decreased cell wall stability (Fig. 9). Diminished cell wall carbohydrate content of P_i_-starved chemostat-grown S. cerevisiae cells was described 4 decades ago, though a mechanism was not proposed (73). Our model predicts that cells lacking Pho84 would be intolerant not just of beta-1,3-glucan synthase inhibition by micafungin (7) but also of genetic or pharmacologic perturbation of beta-1,6-glucan and chitin synthases. The chitin and beta-1,6-glucan contents of cells lacking Pho84 were sharply decreased (Fig. 4). Similarly, wild-type cells starved for P_i_ were hypersensitive to inhibitors of chitin and beta-1,3-glucan synthesis, nikkomycin and micafungin (Fig. 5), consistent with combinatorial effects between depletion of the enzyme substrate and direct enzyme inhibition.

Growth defects of strains that we constructed in which expression of the genes encoding the major beta-1,6-glucan synthase, *KRE6*, and the single essential chitin synthase, *CHS1*, is repressible from *pMAL2* (Fig. 6 and 7) or *tetO* were in agreement with the results of Munro et al. (55) but differed from the findings of Han et al. (18, 59). In some *kre6/pMAL2-KRE6* strains, we also deleted the minor beta-1,6-glucan synthase-encoding gene, *SKN1*, and observed no further effect on the phenotype under the analyzed conditions. While transcription from the repressible promoters we used cannot be completely abrogated (56), residual transcription is not expected to sharpen a loss-of-function-associated growth defect. We speculate that the stronger defects of our *kre6* conditional mutants, compared to the homozygous deletion mutant of Han et al., may be attributable to residual Kre6 activity in our mutants, which lowered the likelihood of suppressor mutation emergence.

Loss of Pho84 exacerbated the growth defects exhibited by *CHS1*- and *KRE6*-depleted strains, especially when P_i_ was not in excess in the medium (Fig. 6 and 7). Conversely, maximally loading cells with P_i_ by prolonged growth in excess P_i_ during depletion of the enzyme in question significantly rescued these growth defects (Fig. 6 and 7), supporting the idea that lack of P_i_ is responsible for synthetic phenotypes of *pho84* with *kre6* or *chs1* mutations.

In agreement with the work of Han et al. (59), we observed a compensatory increase of cell wall chitin (Fig. 8A) in cells depleted of *KRE6*. Han et al. showed that this response depended on intact Mkc1 signaling (59). Absence of Pho84 in *KRE6*-depleted cells abrogated this compensatory response (Fig. 8). Dampened Mkc1 signaling in cells without Pho84 activity (Fig. 1C and Fig. S1) may be one reason for the *pho84^−/−^* mutants’ inability to appropriately upregulate chitin synthase transcription (58, 74). Another reason could be that the concentration of the chitin synthase substrate UDP-GlcNAc was insufficient. The two mechanisms could act together to diminish compensatory chitin synthesis in cells lacking Kre6 as well as Pho84.

Unlike Han et al. (59), we found that very low nikkomycin concentrations further inhibit the growth of *KRE6*-depleted cells, as compensatory chitin synthesis is inhibited (Fig. 8B). This discrepancy could be due to the medium used for assaying the nikkomycin effect: di- and tripeptides, present in YPD but not in the SC medium we used, compete with nikkomycin for uptake into C. albicans cells (75, 76). If novel antifungals could be combined to simultaneously inhibit glucan and chitin synthesis, a potent antifungal effect as well as low toxicity in humans, who lack both targets, could be expected.

Induction of high-affinity P_i_ transporter-encoding genes *PHO84* and *PHO89* in *ex vivo* and *in vivo* models of infection (20–23) shows that during infection, the fungus is challenged with acquiring P_i_, possibly due to alkaline environments in the host (77). While our experimental conditions of prolonged P_i_ excess are apparently not typical for niches inhabited by C. albicans, our results showed that it is limited P_i_ availability that renders depletion of these cell wall biosynthetic enzymes inhibitory to C. albicans growth. They further indicated that during perturbation of biosynthesis of a single cell wall component, P_i_ availability is limiting for production of compensatory cell wall components like chitin.

Investigators examining C. albicans isolates from stool of intensive care unit patients as well as standard laboratory strains hypothesized that phosphate starvation increases hyphal growth and virulence of C. albicans (78). Mice that had undergone partial hepatectomy and were drinking tap water, versus a 25 mM phosphate solution, were more susceptible to cecal injection of a C. albicans suspension in water than in 25 mM phosphate, respectively, and had more C. albicans biofilm on their intestinal mucosa (78). The authors concluded that phosphate starvation had made the C. albicans cells more virulent. Mutants in the phosphate starvation response transcriptional regulator Pho4 were considered to produce more hyphae on low-phosphate than on high-phosphate medium and to be more virulent in a Caenorhabditis elegans model of infection (78). These findings contrast with those of Ikeh et al., who in multiple *ex vivo* and *in vivo* infection models found null mutants in *PHO4* to be attenuated in virulence (38). Since cells without Pho4 have low levels of Pho84 (38), our results align more closely with those of Ikeh et al. (38), as we found attenuated virulence and defective hyphal growth in cells lacking Pho84 (8). The cell wall integrity defects of *pho84* null mutant cells characterized here may also contribute to their virulence attenuation. Differences in experimental conditions may be responsible for the discrepancies between the results reported by these investigators (78) and await analysis in further experiments.

How limited P_i_ supplies and essential intermediate metabolites requiring P_i_ like ATP and phosphoribosyl-pyrophosphate (PRPP) are allocated to different biosynthetic activities of the cell is not known. Decreased phosphomannan, decreased chitin, and decreased beta-1,6-glucan contents suggest that cells lacking P_i_ prioritize use of this essential element for other metabolic processes. P_i_ starvation often limits plant growth: P_i_-starved oats replace up to 70% of their plasma membrane phospholipids with the glycolipid digalactosyldiacylglycerol (79), a process that is reversible upon P_i_ refeeding (80). Fungi including the human pathogen Cryptococcus neoformans also replace cytoplasmic membrane phospholipids with nonphosphoric lipids during P_i_ starvation (81, 82). That the cell envelope—the plasma membrane and cell wall—apparently can function while forgoing a share of P_i_, while DNA polymerase and ribosomes are absolutely dependent on their P_i_ allotment, suggests a regulatory mechanism to assign the available P_i_ to each biosynthetic process. Identification of and interference with this mechanism might lead to a way to disrupt processes required for growth and proliferation of the fungal cell.

Echinocandins and nikkomycin exemplify good tolerability of antifungals whose targets are not conserved in humans (3, 83). Pho84 and beta-1,6-glucan synthases also have no human orthologs. Development and combination of specific small-molecule inhibitors of these targets should potentiate their effects and permit more effective clearance of invasive candidiasis.

## MATERIALS AND METHODS

Detailed descriptions of methods are provided in Text S1 in the supplemental material.

10.1128/mBio.03225-19.1 TEXT S1 Detailed methods. Detailed descriptions of the methods used in this study are provided in order to facilitate reproducibility. Download Text S1, PDF file, 0.1 MB. Copyright © 2020 Liu et al. 2020 Liu et al. This content is distributed under the terms of the Creative Commons Attribution 4.0 International license.

### Strains and culture conditions.

C. albicans strains used are shown in Table S2A. Strains were constructed as described in reference 56, using plasmids shown in Table S2B and oligonucleotides shown in Table S2C, using sequences obtained from the Candida Genome Database (84). To minimize phenotypic artifacts originating from genomic events unrelated to the targeted introduced mutations, all genotypes examined were constructed from at least 2 independently engineered heterozygous strains. Experiments with defined ambient P_i_ concentrations were performed in media based on yeast nitrogen base (YNB) 0 P_i_ (ForMedium Ltd., Norfolk, United Kingdom) with added KH_2_PO_4_ to stated concentrations. P_i_ starvation was induced as described in reference 36. Other media were used as previously indicated (56).

10.1128/mBio.03225-19.9 TABLE S2 Molecular biologic reagents. Strains, plasmids, oligonucleotides, and antibodies used in this study are listed and described. Download Table S2, PDF file, 0.1 MB. Copyright © 2020 Liu et al. 2020 Liu et al. This content is distributed under the terms of the Creative Commons Attribution 4.0 International license.

### Western blots.

Cell lysis and Western blotting were performed as described in reference 30. Antibodies used are listed in Table S2D. At least three biological replicates were obtained.

### Alcian blue staining assay.

The standard curve and alcian blue binding were determined as in the work of Hobson et al. (34).

### Transmission electron microscopy.

Each strain was inoculated in standard SC to an optical density at 600 nm (OD_600_) of 0.1 and grown for 15 h. Cells were then prepared for TEM and analyzed as described in reference 60 with minor modifications.

### Intracellular ATP measurement.

Cells cultured overnight in YPD medium with a starting OD_600_ of 0.2 were washed with sterile water and lysed. Intracellular ATP levels were measured using the CellTiter-Glo luminescent cell viability assay (Promega; catalog no. G7570), and the results were normalized to the protein concentration. The standard curve was prepared using ATP disodium salt hydrate (Sigma; catalog no. A6419).

### Metabolomics.

Metabolites were measured as described in reference 39.

### Hyphal morphogenesis assay.

Cells were resuspended in 0.9% NaCl to an OD_600_ of 0.1. Variations between single colonies as well as colony density effects were minimized by spotting 3-μl cell suspensions at 6 equidistant points, using a template, around the perimeter of an agar medium plate as described in reference 56. Spider medium and RPMI 1640 with glutamine, without sodium bicarbonate (Gibco 31800-022), were used.

### Chitin measurement.

Cells were grown for 8 h in SC low (0.22 mM) or excess (11 mM) P_i_ after pregrowth of 3 days’ passages in 2× SC-P_i_ 2% maltose. At the end of pregrowth (time zero) and at 8 h, cells were formaldehyde fixed, washed in phosphate-buffered saline (PBS) before staining with calcofluor white, and then washed extensively in 0.9% NaCl. After sonication, fluorescence intensities of cells were measured by flow cytometry of ≥10^5^ events.

### *PHO84* promoter induction analysis.

Cells of genotype *PHO84/pPHO84-GFP-NAT1-PHO84* were grown in YPD with additional 10 mM P_i_ for 16 h and washed three times with 0.9% NaCl, and OD_600_ was adjusted to 0.01 in SC with increasing concentrations of KH_2_PO_4_. OD_600_ and GFP signal were recorded every 30 min.

### Beta-1,6-glucan measurement.

Cell wall glucans were extracted, adapting the method of Gilbert et al. (85), by alkali extracting crude cell lysate with 0.75 N NaOH at 75°C for an hour. Supernatants containing alkali-soluble glucans were stored at −80°C until analysis by ELISA. Insoluble pellets were digested with chitinase and Zymolyase in 100 mM K_2_HPO_4_-KH_2_PO_4_ buffer (pH 6.0) for 72 h at 37°C followed by 1 h at 45°C. Supernatants of these digests containing alkali-insoluble glucans were stored at −80°C until analysis by ELISA. A competition ELISA using an anti-beta-1,6-glucan antibody (48) was performed with modifications as described in reference 48.

### Statistical analysis.

Statistical analysis was performed by unpaired Student’s *t* test in Prism 7 GraphPad (GraphPad Software, Inc., CA, USA). For metabolomics, MetaboAnalyst (40) was used for analysis including principal-component analysis, heat maps, and pathway analysis.
