# Supplementary figures and images for: Phosphoric Metabolites Link Phosphate Import and Polysaccharide Biosynthesis for Candida albicans Cell Wall Maintenance
Source: mBio. 2020 Mar 17;11(2):e03225-19. doi: 10.1128/mBio.03225-19 (PMC7078483; doi:10.1128/mBio.03225-19)

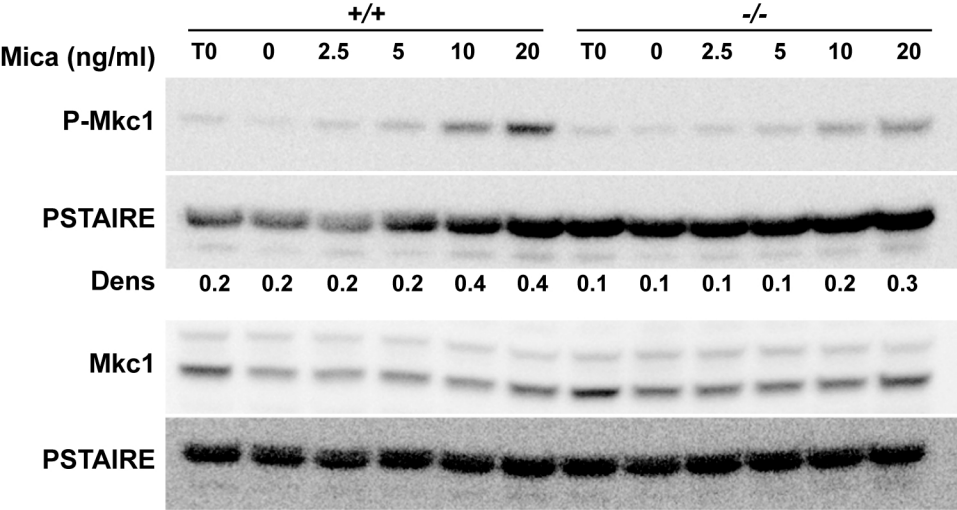

Supplement: FIG S1 [file mBio.03225-19-sf001.pdf]

**A**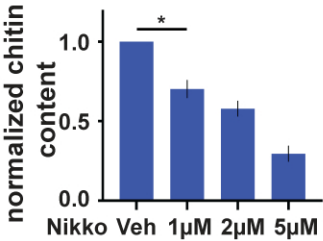**B**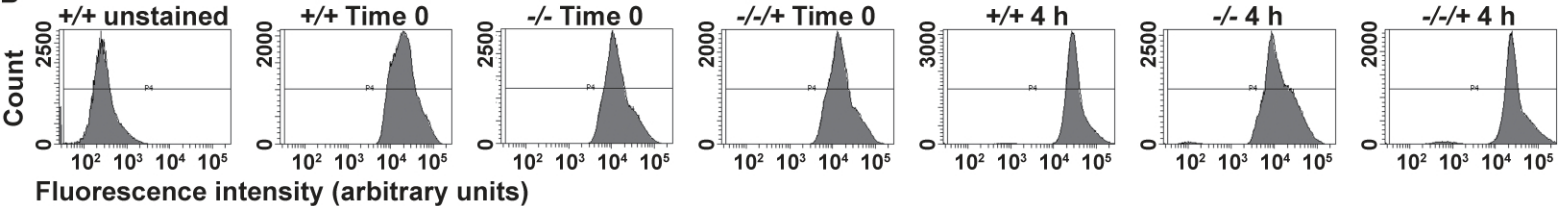

Supplement: FIG S2 [file mBio.03225-19-sf002.pdf]

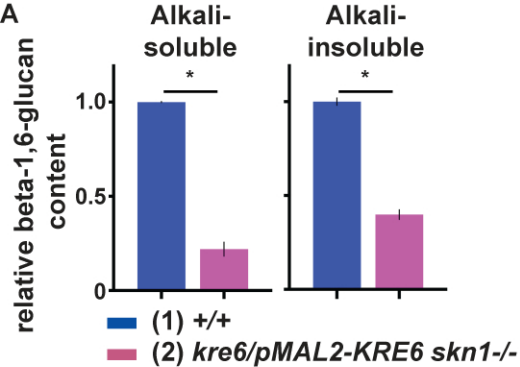

beta-1,6-glucan dot blot

2-fold dilution

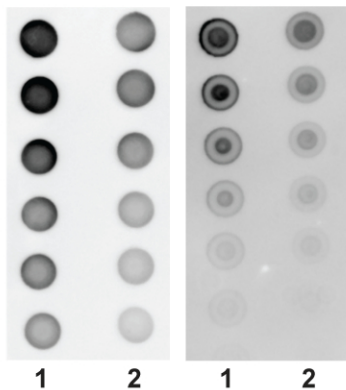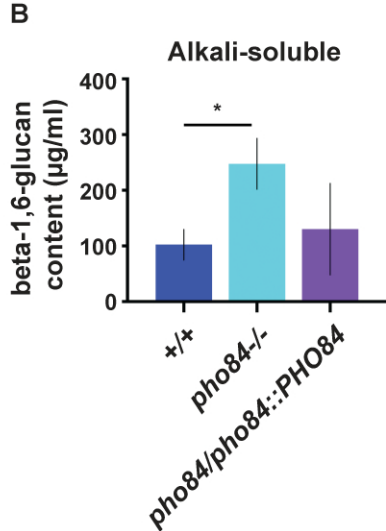

Supplement: FIG S3 [file mBio.03225-19-sf003.pdf]

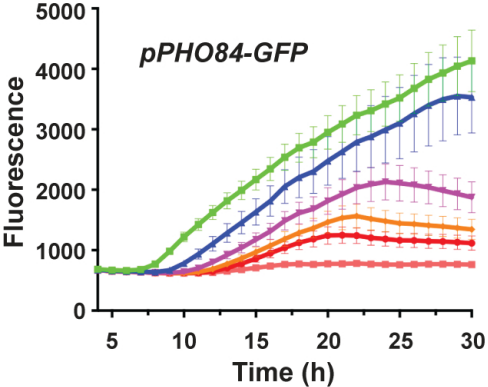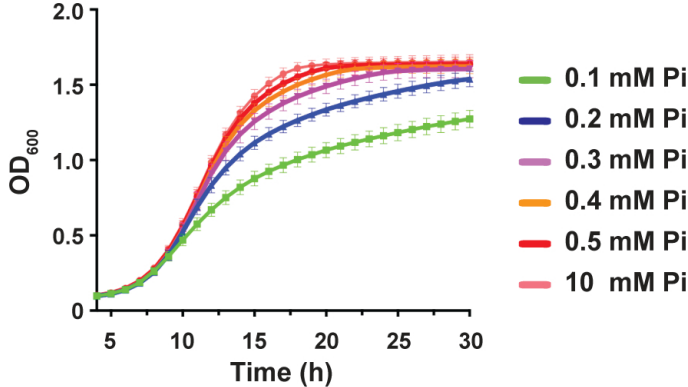

Supplement: FIG S4 [file mBio.03225-19-sf004.pdf]

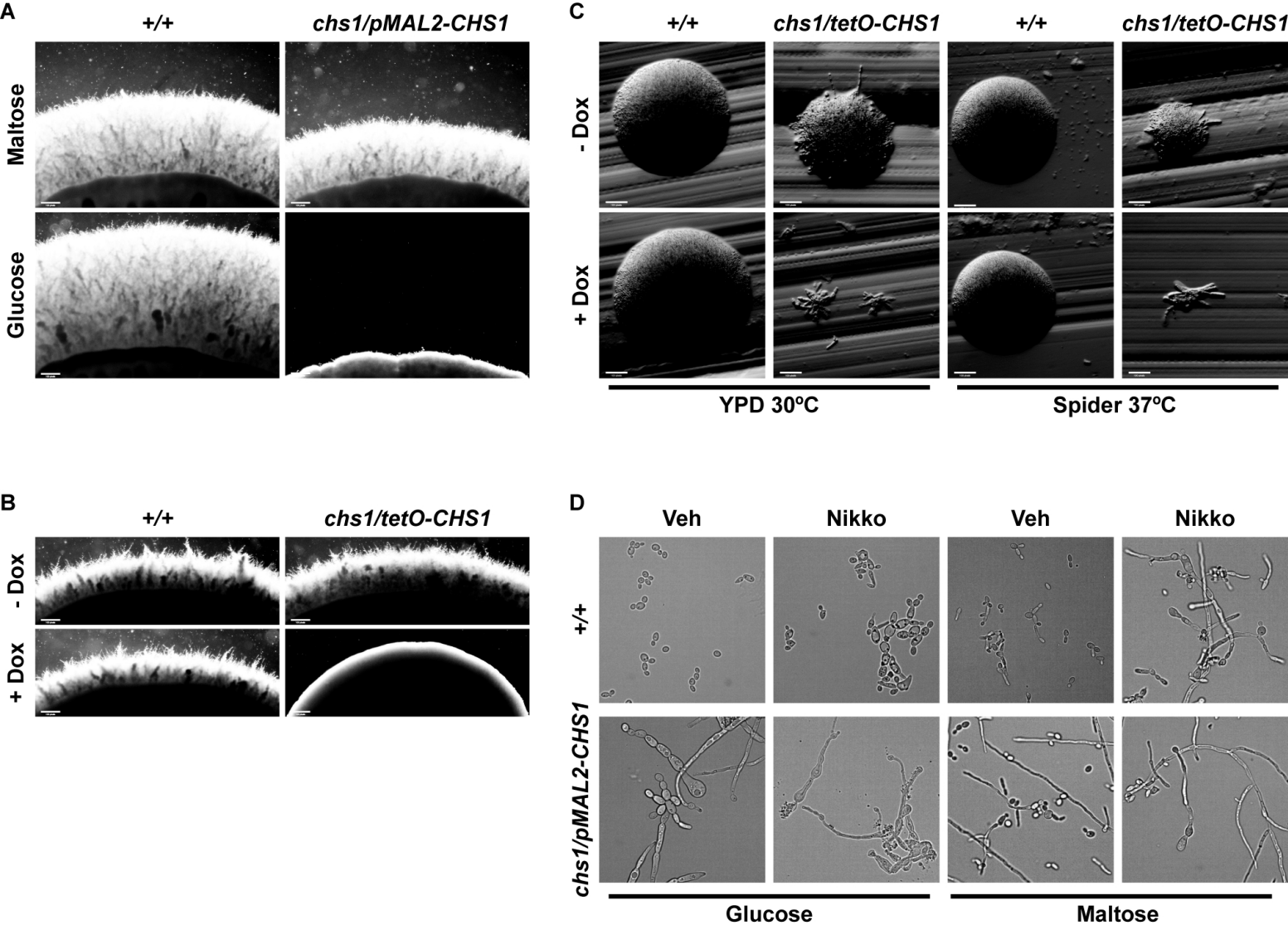

Supplement: FIG S5 [file mBio.03225-19-sf005.pdf]

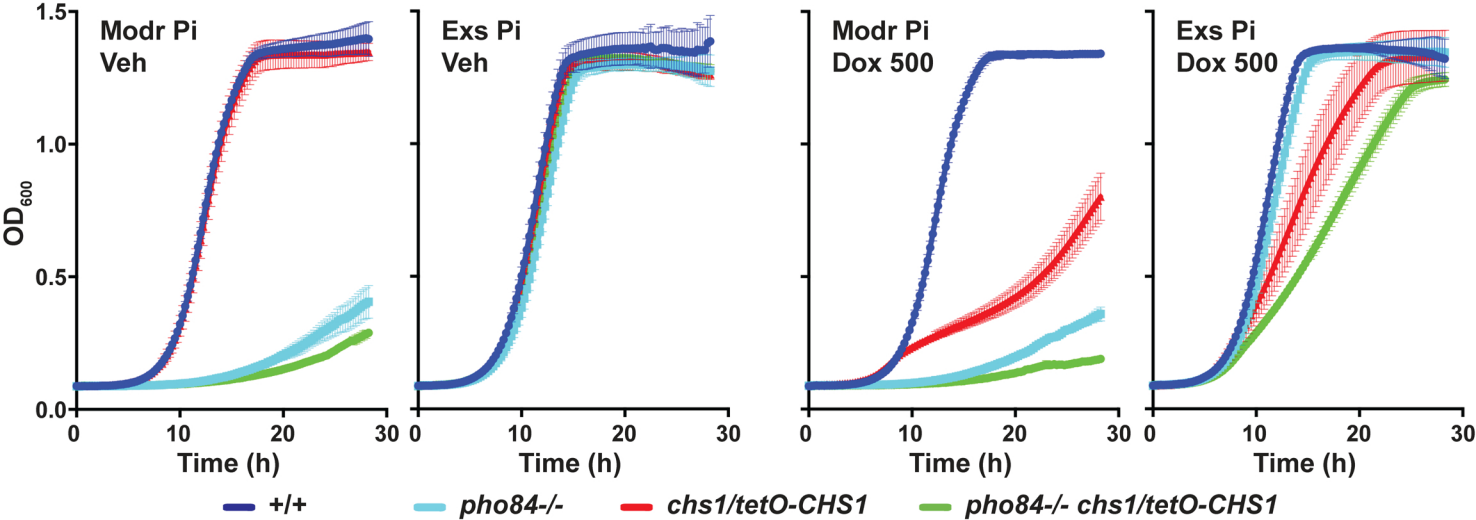

Supplement: FIG S6 [file mBio.03225-19-sf006.pdf]
