# Supplementary material for: Phosphoric Metabolites Link Phosphate Import and Polysaccharide Biosynthesis for Candida albicans Cell Wall Maintenance
Source: mBio. 2020 Mar 17;11(2):e03225-19. doi: 10.1128/mBio.03225-19 (PMC7078483; doi:10.1128/mBio.03225-19)
Supplement: TABLE S1 [file mBio.03225-19-st001.pdf]

Table S1. Metabolites of significantly different abundance between *pho84-/-* and wild type cells.

***pho84* null/wild type in 0.22 mM Pi**			
	**FC**	**log2(FC)**	**p.adjusted**
allantoin	0.040402	-4.6294	3.14E-05
cytidine	16.265	4.0237	6.04E-05
a-ketoglutarate	4.949	2.3071	6.78E-05
uracil	20.496	4.3573	6.78E-05
aconitate	18.359	4.1984	7.58E-05
ornithine	0.31017	-1.6889	9.10E-05
S-adenosyl-L-homocysteine-nega	0.016334	-5.936	0.00010768
xanthosine	10.271	3.3605	0.00010768
cytosine	15.642	3.9673	0.00014931
dCDP-nega	0.044387	-4.4937	0.00014931
histidine	7.046	2.8168	0.00014931
trehalose-sucrose	87.873	6.4573	0.00014931
UMP	0.18964	-2.3987	0.00014931
dTMP	0.032259	-4.9541	0.00014978
guanosine	8.5572	3.0971	0.00014978
S-adenosyl-L-homoCysteine-posi	0.011215	-6.4785	0.00015411
CMP	0.078521	-3.6708	0.0001767
phenylpyruvate	0.015261	-6.034	0.0001767
coenzyme A-posi	0.17741	-2.4948	0.00018045
coenzyme A-posi1	0.19274	-2.3753	0.00018917
isocitrate	5.1245	2.3574	0.00022474
Imidazoleacetic acid	15.689	3.9716	0.00023789
orotate	2.3986	1.2622	0.00030685
citrulline	0.11782	-3.0853	0.00031049
N-acetyl-glutamine	2.4623	1.3	0.00031767
Carbamoyl phosphate	0.059699	-4.0662	0.00034619
Cellobiose	5.5992	2.4852	0.00034619
GTP-nega	0.045634	-4.4538	0.00034619
guanine	3.7781	1.9177	0.00034619
hypoxanthine	2.4261	1.2787	0.00034619
taurine	6.5313	2.7074	0.00034619
glutamine	3.7085	1.8908	0.00036325
Methionine sulfoxide	0.08586	-3.5419	0.00043664
3-phosphoglycerate	0.040431	-4.6284	0.00052767
anthranilate	2.0315	1.0226	0.00052767
glutathione disulfide-nega	2.9756	1.5732	0.00052767
guanosine 5-diphosphate,3-diphosphate	0.039778	-4.6519	0.00052767
phosphoenolpyruvate	0.095344	-3.3907	0.00052767
oxaloacetate	5.4815	2.4546	0.00061397
dihydroxy-acetone-phosphate	5.3781	2.4271	0.0006369
tryptophan	5.5028	2.4602	0.00072306

5-methoxytryptophan	4.027	2.0097	0.00074365
dTDP-nega	0.021206	-5.5594	0.00074365
dATP-nega	0.14312	-2.8047	0.00076604
dCMP	0.12106	-3.0462	0.00078067
NADH	8.3553	3.0627	0.00080057
adenine	3.1454	1.6533	0.00093174
Glycerophosphocholine	0.057117	-4.1299	0.00097249
inosine	3.664	1.8734	0.00097249
glutathione disulfide-posi	3.2939	1.7198	0.00098563
tyrosine	3.0281	1.5984	0.0012842
D-glyceraldehdye-3-phosphate	5.047	2.3354	0.001332
Uric acid	7.7848	2.9607	0.001332
Acetylcarnitine DL	6.9729	2.8018	0.0013367
dTMP-nega	0.067314	-3.893	0.0013367
homocysteine	0.46439	-1.1066	0.0013367
S-adenosyl-L-methionine	0.082765	-3.5948	0.0013367
UDP-N-acetyl-glucosamine	0.12642	-2.9837	0.0013367
3-hydroxy-3-methylglutaryl-CoA-nega	0.066132	-3.9185	0.0013678
D-glucarate	3.5172	1.8144	0.0013678
5-phosphoribosyl-1-pyrophosphate	0.012935	-6.2726	0.0014299
2,3-dihydroxybenzoic acid	4.292	2.1016	0.0017393
homocysteic acid	6.0869	2.6057	0.0017496
3-hydroxybuterate	4.413	2.1418	0.0017749
pyruvate	2.2134	1.1463	0.0017749
citrate	2.5656	1.3593	0.0019009
malate	2.3248	1.2171	0.0019019
UDP-nega	0.04168	-4.5845	0.0020831
2-deoxyglucose-6-phosphate	0.33792	-1.5652	0.0021086
quinolinate	3.0169	1.5931	0.0022146
UDP-D-glucose	0.16349	-2.6128	0.0022146
dehydroascorbic acid	3.7037	1.889	0.0022528
deoxyadenosine	0.074291	-3.7507	0.0022528
hexose-phosphate	0.4641	-1.1075	0.0022528
ADP-nega	0.12091	-3.048	0.0023643
dGDP-nega	0.12091	-3.048	0.0023643
UTP-nega	0.061988	-4.0119	0.0024336
2-oxo-4-methylthiobutanoate	52.512	5.7146	0.0025326
Ng,NG-dimethyl-L-arginine	2.3151	1.2111	0.0025977
Atrolactic acid	11.351	3.5048	0.0034416
Methylcysteine	3.1644	1.6619	0.0034622
xanthine	3.1256	1.6441	0.0035048
aminoimidazole carboxamide ribonucleotide	0.13195	-2.9219	0.0038246
CDP-ethanolamine	0.065973	-3.922	0.0040087
Citraconic acid	2.0873	1.0616	0.0040446
dUTP-nega	0.2869	-1.8014	0.0045111
IDP-nega	0.1118	-3.1611	0.0045111
Deoxycholic acid	3.9582	1.9848	0.0049187
O8P-O1P	0.10187	-3.2952	0.0049187

UDP-D-glucuronate	3.953	1.9829	0.0049187
sn-glycerol-3-phosphate	0.14551	-2.7808	0.0050572
CDP-nega	0.19981	-2.3233	0.0052038
methionine	0.42067	-1.2492	0.0052719
Geranyl-PP	0.037885	-4.7222	0.0054001
carnitine	2.1092	1.0767	0.0056029
dimethylglycine	0.24819	-2.0105	0.0059677
glycerate	3.5136	1.813	0.0060796
glutamate	3.2973	1.7213	0.0068881
dTTP-nega	0.071589	-3.8041	0.0068961
nicotinamide	0.26599	-1.9106	0.0068961
6-phospho-D-gluconate	0.10918	-3.1953	0.0069544
orotidine-5-phosphate	5.5537	2.4735	0.007526
fructose-1,6-bisphosphate	0.13146	-2.9273	0.0075665
4-Pyridoxic acid	2.1717	1.1188	0.0076503
succinyl-CoA-posi	0.35317	-1.5016	0.0076503
GDP-nega	0.077609	-3.6876	0.008032
SBP	0.26448	-1.9188	0.0080568
dAMP	0.20427	-2.2915	0.0082617
glucose-1-phosphate	0.49859	-1.0041	0.0095968
N-acetyl-glucosamine	4.1855	2.0654	0.010297
N-carbamoyl-L-aspartate	2.9407	1.5562	0.010297
CDP-choline	0.052741	-4.2449	0.010639
acetylphosphate	0.34488	-1.5358	0.010851
glucosamine	5.5759	2.4792	0.011884
glycine	0.18573	-2.4287	0.011961
glutathione	2.0759	1.0537	0.013059
ATP-nega	0.18394	-2.4427	0.013111
dGTP	0.18394	-2.4427	0.013111
N-carbamoyl-L-aspartate-nega	0.36009	-1.4736	0.013473
nicotinate	4.3186	2.1106	0.013473
biotin	2.9584	1.5648	0.013726
hydroxyproline	0.34237	-1.5464	0.014091
ethanolamine	5.0973	2.3497	0.015694
Pyrophosphate	0.27556	-1.8596	0.015775
4-phosphopantothenate	0.20137	-2.3121	0.017647
hydroxyphenylpyruvate	3.0201	1.5946	0.018717
coenzyme A-nega	0.099176	-3.3339	0.01898
folate	2.0272	1.0195	0.019401
dUMP-nega	0.12421	-3.0092	0.019816
creatine	6.2046	2.6333	0.019875
ribose-phosphate	0.45724	-1.129	0.024468
CTP-nega	0.14413	-2.7945	0.026343
Urea	0.35241	-1.5047	0.027388
allantoate	0.34131	-1.5508	0.02783
2-dehydro-D-gluconate	3.7485	1.9063	0.028256
Diiodothyronine	7.382	2.884	0.029962
xanthosine-5-phosphate	4.1266	2.045	0.033932

Cystine	4.6019	2.2022	0.034141
Thiamine pyrophosphate	0.33502	-1.5777	0.034572
NAD_nega	0.42679	-1.2284	0.035408
pyridoxine	0.44134	-1.18	0.036494
Guanidoacetic acid	7399.4	12.853	0.037222
D-erythrose-4-phosphate	2.222	1.1519	0.039076
NAD_posi	0.46471	-1.1056	0.040489
adenosine 5-phosphosulfate	0.18284	-2.4513	0.042405
thiamine-phosphate	9.0747	3.1818	0.04614
Pyridoxamine	7.9818	2.9967	0.046166
S-adenosyl-L-methioninamine	4.8342	2.2733	0.046166
spermidine	4.2389	2.0837	0.047587
***pho84* null/wild type in 11 mM Pi**			
	FC	log2(FC)	p.ajusted
5-phosphoribosyl-1-pyrophosphate	0.096386	-3.375	0.0001532
Ascorbic acid	0.004486	-7.8004	0.0001532
N-carbamoyl-L-aspartate-nega	0.096127	-3.3789	0.0001532
orotate	16.808	4.0711	0.0001532
allantoate	0.099225	-3.3332	0.00018163
N-carbamoyl-L-aspartate	0.16159	-2.6296	0.00018163
citrate	7.7874	2.9611	0.00053737
dihydroorotate	0.097491	-3.3586	0.00053737
citrate-isocitrate	3.6146	1.8538	0.00055148
isocitrate	21.316	4.4139	0.00057659
pyruvate	11.677	3.5455	0.00059731
aconitate	20.329	4.3455	0.00074542
shikimate	0.14751	-2.7611	0.00087945
homocysteic acid	2.0967	1.0681	0.0017237
2-oxobutanoate	5.5625	2.4757	0.0027495
phosphoenolpyruvate	0.48634	-1.04	0.0027495
L-arginino-succinate	0.3506	-1.5121	0.0029383
Phenylpropiolic acid	3.3255	1.7336	0.0029645
cytidine	3.0792	1.6226	0.0030524
xanthosine	3.4986	1.8068	0.0030711
cytosine	2.5123	1.329	0.0033458
phenylpyruvate	2.9843	1.5774	0.0040184
guanosine	3.5022	1.8082	0.0043891
O8P-O1P	0.4388	-1.1884	0.0046627
CDP-ethanolamine	0.42506	-1.2343	0.005099
sn-glycerol-3-phosphate	0.43324	-1.2068	0.0067375
UDP-nega	0.48955	-1.0305	0.0086487
inosine	2.1612	1.1119	0.011046
Citraconic acid	7.1519	2.8383	0.011942
3-phosphoglycerate	0.44	-1.1844	0.013922
Carbamoyl phosphate	0.39954	-1.3236	0.019209
deoxyguanosine	2.553	1.3522	0.019209
Acetyllysine	0.45755	-1.128	0.020772

Pyrophosphate	0.36232	-1.4647	0.020772
coenzyme A-posi	0.38883	-1.3628	0.021741
dCMP	0.49564	-1.0126	0.027542
3-hydroxybuterate	3.4758	1.7974	0.033727
SBP	17.703	4.146	0.038308
Hydroxyphenylacetic acid	2.3195	1.2138	0.03986
