# Supplementary material for: Phosphoric Metabolites Link Phosphate Import and Polysaccharide Biosynthesis for Candida albicans Cell Wall Maintenance
Source: mBio. 2020 Mar 17;11(2):e03225-19. doi: 10.1128/mBio.03225-19 (PMC7078483; doi:10.1128/mBio.03225-19)
Supplement: TEXT S1 [file mBio.03225-19-s0001.pdf]

Text S1. Detailed Methods.

Strains and culture conditions.
For growth curves in liquid media, cells were subjected to described growth conditions, or as a default condition grown on a YPD plate for 2 days after revival from stock held at -80°C.

For growth curves, cells were scraped from the plate and washed once in 0.9% NaCl and diluted to OD_{600} 0.01, in 150 μl medium in flat bottom 96-well dishes. OD_{600} readings were obtained every 15 min in a BioTek™ Synergy™ 2 Multi-Mode Microplate Reader (Winooski, VT, USA). At least 3 biological replicates were obtained on different days. Standard deviations of 3 technical replicates, representing separate wells, were calculated and graphed in Graphpad Prism.

For cell dilution spot assays, cells grown on a YPD plate for 2 days after revival from stock were washed with and diluted in 0.9% NaCl in fivefold steps beginning at OD_{600} 0.5. They were spotted onto agar media from round bottom 96 well plates using a VP407 fixed pin replicator from V&P Scientific (San Diego, CA) which delivers ~1.5 μl drops.

Western Blots.
Cells cultured overnight in YPD medium were diluted into YPD or SC with 0.2 mM Pi at OD_{600} 0.2. After 4 hours, micafungin was added to the indicated concentrations and cultures were incubated for 90 min. Cell lysis and Western blotting were performed as described in (1). Antibodies used are listed in Table S5. At least three biological replicates were obtained. For densitometry, ImageJ (imagej.net/welcome) software was used to quantitate signals obtained on a KODAK Image Station 4000MM.

Alcian Blue Staining assay.
Cells were patched from -80°C stock onto a YPD plate and incubated at 30°C for 15 hours. Cells were then inoculated into SC with 1 mM, 7.3 mM and 11 mM Pi at OD_{600} 0.2 and shaken for 15 hours. Cells were washed twice with sterile water and 5 OD_{600} cells were resuspended in 1 ml Alcian Blue (Alfa Aesar, J60122; 30 µg/ml in 0.02 M HCl) in triplicate, then incubated 10 min at room temperature. Cells were pelleted and the OD_{620} of 100 µl supernatant was measured in a 96-well plate using a Tecan Infinite M200 plate reader (Tecan Trading AG, Switzerland). The standard curve and Alcian blue concentration were determined as in Hobson et al. (2), i.e. Alcian Blue binding (pg/cell) was calculated according to the formula: $x = [(u-v) \div n] \times 10^6$ where x = Alcian Blue bound (pg/cell), u = original Alcian Blue concentration (µg/ml), v = final Alcian Blue concentration (µg/ml), and n = number of cells stained.

Transmission Electron Microscopy.
Each strain was inoculated into SC medium to an OD_{600} of 0.1 and grown for 15 h. Cells were then prepared for TEM and analyzed as described in (3) with minor modifications. Cells were collected and washed with mQ-water, fixed by high-pressure freezing using a Leica EM PACT2 and freeze substituted using a Leica AFS 2. The thickness of the inner cell wall layer (glucan

and chitin) and outer fibrillar layer was determined from TEM pictures using ImageJ software. Measurements were obtained from 50 cells of each strain that were sectioned so that cell wall thickness was even all around the cell periphery.

Metabolomics.

C. albicans cells were grown in SC-Pi for 3 days at starting OD_{600} 0.2 and inoculated into fresh medium every day as in (4). Harvested cells were washed with sterile water three times and then incubated in SC medium with 0.22 mM or 11 mM KH_2PO_4 for 4 hours. Collected cells were washed with sterile water three times. The cell pellets were resuspended in 500 µl 80% methanol (-80°C) on ice and transferred to -80°C for 40 min. After centrifugation, the supernatant was collected and dried by Savant SpeedVac DNA 110 (GMI, Minnesota, USA). This extraction was repeated for a total of three times. The dried pellets were stored at -80°C until metabolomics analysis (5).

For LC-MS/MS, metabolite pellets were resuspended in 20 µl LC-MS-grade water, and 5 µl was injected over a 15-min gradient using a 5500 QTrap triple quadrupole mass spectrometer (AB Sciex) coupled to a Prominence ultrafast LC (UFLC) high-performance liquid chromatography (HPLC) system (Shimadzu) via SRM (selected reaction monitoring) of a total of 287 SRM transitions using positive- and negative-polarity switching corresponding to 258 unique endogenous water-soluble metabolites. The dwell time was 3 ms per SRM, resulting in ~10 to 14 data points acquired per detected metabolite. Samples were separated using an Amide XBridge HPLC hydrophilic interaction liquid chromatographic (HILIC) column (3.5 µm; 4.6-mm inner diameter by 100-mm length; Waters) at 300 µl/min. Gradients were run starting from 85% buffer B (HPLC-grade acetonitrile) to 40% B from 0 to 5 min and 40% B to 0% B from 5 to 16 min, 0% B was held from 16 to 24 min, the gradient was run from 0% B to 85% B from 24 to 25 min, and 85% B was held for 7 min to reequilibrate the column. Buffer A was comprised of 20 mM ammonium hydroxide-20 mM ammonium acetate (pH 9.0) in 95:5 water-acetonitrile. Peak areas from the total ion current for each metabolite SRM transition were integrated using MultiQuant version 2.1 software (AB Sciex) via the MQ4 peak integration algorithm using a minimum of 8 data points with a 20-s retention time window.

Hyphal morphogenesis assay.

Cells were revived from frozen stocks on solid YPD overnight, washed and resuspended in 0.9% NaCl to OD_{600} 0.1. Variations between single colonies and colony density effects were minimized by spotting 3 µl cell suspension at 6 equidistant points, using a template, around the perimeter of an agar medium plate as in (6). Spider medium and RPMI1640 with glutamine, without sodium bicarbonate (Gibco 31800-022) were used. RPMI 1640 medium was buffered to pH 5.5 with 165 mM MOPS and 2 g/L sodium bicarbonate, and the desired carbon source (maltose or glucose) was added to a total of 2%, in addition to 0.2% glucose contained in commercially available formulations of RPMI 1640. All panels shown represent ≥3 biological replicates.

***PHO84* promoter induction assay.**

Cells of genotype *PHO84/pPHO84-GFP-NAT1-PHO84* were grown in YPD liquid medium with additional 10 mM Pi for 16 h. After 3 washes with 0.9% NaCl, the OD_{600} was adjusted to 0.01 in SC with increasing concentrations of KH_2PO_4 (0.1, 0.2, 0.3, 0.4, 0.5 and 10 mM) and 50 μl/well of 8 technical replicates for each condition were inoculated into a black 384 well plate with transparent bottom. During incubation at 30°C, OD_{600} and GFP signal (Ex 485/20 nm; Em 528/20 nm) were recorded every 30 min at gain 50 in a Synergy 2 BioTek Plate reader. Readings were graphed in Graphpad Prism.

Chitin measurement.
Cells were fixed in the growth medium by adding 50 μl 36.5%-38% formaldehyde (Sigma, F-8775) per ml cell culture (formaldehyde final concentration ~1.8%) and incubating at room temperature for 30 min. Fixed cells were washed twice with PBS before staining with 10 μg/ml Calcofluor White (Sigma, F-3543-1G) in water for 4 min at room temperature. Stained cells were washed five times with 0.9% NaCl with a 30 minute room temperature incubation step after the 3rd wash, then resuspended in 500 μl 0.9% NaCl and stored at 4ºC before processing for flow cytometry.

Calcofluor stained cells were sonicated (20 sec x 2, microtip, setting 3.5) before flow cytometry (BD LSRFortessa™ Cell Analyzer, BD Biosciences, 647177). Chitin staining was detected using a violet laser (405 nm) and signal collected with channel BV421 (fluorescence filter 450/50 nm) and channel BV510 (fluorescence filter set 505LP, 525/50 nm).

Beta-1,6-glucan measurement.
Glucan extraction (modified from (7)): cells were recovered from -80°C on YPD or YPM plates and grown under desired conditions. Harvested cells were washed once and resuspended in 0.5 ml ice-cold H_2O and transferred to screw-cap tubes pre-loaded with 0.5 ml silica beads (BioSpec Products, 11079101z). Cells were lysed with bead beating for 30 sec x5 times at 4ºC, with 3 min intermittent incubation on ice. Lysate was removed from beads and protein content was determined using Pierce BCA protein assay kit (Thermo Scientific, 23227). Crude cell lysate volume equivalent to an 8 µg protein content was alkali-extracted with 50 µl of 1.5 N NaOH (total volume of 100 µl) at 75ºC for 1 h. Samples were centrifuged 5 min at 11,000xg and the supernatants containing alkali-soluble glucans were collected. The alkali-insoluble pellets were washed with 500 µl water and resuspended in 100 µl of 100 mM K_2HPO_4-KH_2PO_4 buffer (pH 6.0) containing 0.05 U chitinase from *Streptomyces griseus* (Sigma, C-6137-5UN) and 30 U Zymolyase (Zymo Research, E1006), and incubated at 37ºC for 72 h followed by 45ºC for 1 h. After incubation, samples were centrifuged 5 min at 11,000xg and the supernatants containing glucans released by enzymatic digestion were collected.

Dot blot: Cells of indicated genotypes were inoculated into 2xSC 0.2 mM Pi 2% glucose medium and grown for 20 h. Beta-1,6-glucan was extracted and 2 μl of serial two-fold dilutions of the alkali-soluble and -insoluble fractions were spotted onto a nitrocellulose membrane and dried completely. The membrane was blocked with 5% skimmed milk in PBST (Phosphate Buffer Saline, 0.5% Tween 20) at room temperature for 1 h, washed 3 times with PBST, then

incubated with primary anti-pustulan antibody (8) (1:1000 in PBST, 1% skimmed milk) at 4ºC overnight. After 3 washes with PBST, the membrane was incubated with secondary antibody (Cell Signaling Technology, 7074S; 1:1000 in PBST, 1% skimmed milk) at room temperature for 1 h, then washed 3 times with PBST before chemiluminescent substrate (Thermo Scientific, 34579) was added, and signal was read using a Kodak Image Station (Kodak, 4000MM).

ELISA (modified from (8)): Wells of Nunc™ MicroWell™ 96-well plates (Thermo Scientific, #80040LE) were coated by adding 200 µl purified beta-1,6- glucan (pustulan; InvivoGen, #tlrl-pst) at 2 µg/ml in 0.5 M sodium bicarbonate-sodium carbonate buffer, pH 9.6 to each well, and incubating overnight at 4°C. Plates were then washed 4 times with PBS containing 0.05% (vol/vol) Tween 20 (PBST) and then blocked with 200 µl 1% BSA (Equitech-Bio, Inc, BAC61-0100) in PBST (PBST-BSA) for 2 h at room temperature. Pustulan standards (eight 3-fold dilutions; 2,000 to 0 ng/ml in PBST-BSA) or glucan samples were mixed with an equal volume of anti-pustulan antibody (8) diluted to 1:150,000 in PBST-BSA and pre-incubated for 30 min at 37°C with mixing. After 4 washes with PBST, 100 µl of the glucan samples or standards-plus-antibody mixture was applied to the pustulan-coated wells and incubated for 1 h at room temperature. After 4 washes with PBST 100 µl of secondary goat anti-rabbit HRP-conjugated antibody (Cell Signaling Technology, 7074S) (1:20,000 in PBST-BSA) was added and plates were incubated for 1 h at room temperature. Wells were washed 6 times before adding 100 µl of TMB substrate (Invitrogen, #00-4201-56). After 5 min, the reaction was terminated by adding 50 µl 2N H_2SO_4. The absorbance at 450 nm was measured in a VersaMax plate reader (Molecular Devices, Sunnyvale, CA), and standards were plotted using the 4-parameter curve-fitting setting of the SoftMax Pro software.

Statistical analysis.
Statistical analysis was performed by unpaired Student's *t*-test in Prism 7 Graphpad (GraphPad Software, Inc., CA, USA). For metabolomics, MetaboAnalyst (9) was used for analysis including performing principal component analysis (PCA), creating heat maps and pathway analysis. Metabolomics data was normalized to the reference (WT) for each compound on a per compound basis. Missing values below the threshold level were imputed with the half of the minimum value obtained for the compound within all groups. Each biochemical value was then transformed using generalized logarithm transformation and paretoscaling for distributional assumptions of chemometrics. Statistical analyses were performed using in house scripts, R statistical packages and MetaboAnalyst 3.0 (10-12). Paired *t*-tests with $p \leq 0.05$ and those that have >2 fold greater intensity were considered significant. Principal component analyses were performed using prcomp, biplot and vegan package. Multiple comparisons were accounted by estimating the false discovery rate (FDR) using q-values (13). Pathway reconstruction was performed using Cytoscape v.3.4 and MetScape plugin v.3.1.2

References for Detailed Methods.

1. Chowdhury T, Köhler JR. 2015. Ribosomal protein S6 phosphorylation is controlled by TOR and modulated by PKA in Candida albicans. Mol Microbiol 98:384-402.

2. Hobson RP, Munro CA, Bates S, MacCallum DM, Cutler JE, Heinsbroek SE, Brown GD, Odds FC, Gow NA. 2004. Loss of cell wall mannosylphosphate in Candida albicans does not influence macrophage recognition. J Biol Chem 279:39628-35.
3. Hall RA, Bates S, Lenardon MD, Maccallum DM, Wagener J, Lowman DW, Kruppa MD, Williams DL, Odds FC, Brown AJ, Gow NA. 2013. The Mnn2 mannosyltransferase family modulates mannoprotein fibril length, immune recognition and virulence of Candida albicans. PLoS Pathog 9:e1003276.
4. Popova Y, Thayumanavan P, Lonati E, Agrochao M, Thevelein JM. 2010. Transport and signaling through the phosphate-binding site of the yeast Pho84 phosphate transceptor. Proc Natl Acad Sci U S A 107:2890-5.
5. Yuan M, Breitkopf SB, Yang X, Asara JM. 2012. A positive/negative ion-switching, targeted mass spectrometry-based metabolomics platform for bodily fluids, cells, and fresh and fixed tissue. Nat Protoc 7:872-81.
6. Shen J, Cowen LE, Griffin AM, Chan L, Köhler JR. 2008. The Candida albicans pescadillo homolog is required for normal hypha-to-yeast morphogenesis and yeast proliferation. Proc Natl Acad Sci U S A 105:20918-23.
7. Gilbert NM, Donlin MJ, Gerik KJ, Specht CA, Djordjevic JT, Wilson CF, Sorrell TC, Lodge JK. 2010. KRE genes are required for beta-1,6-glucan synthesis, maintenance of capsule architecture and cell wall protein anchoring in Cryptococcus neoformans. Mol Microbiol 76:517-34.
8. Kottom TJ, Hebrink DM, Jenson PE, Gudmundsson G, Limper AH. 2015. Evidence for Proinflammatory beta-1,6 Glucans in the Pneumocystis carinii Cell Wall. Infect Immun 83:2816-26.
9. Xia J, Wishart DS. 2011. Web-based inference of biological patterns, functions and pathways from metabolomic data using MetaboAnalyst. Nat Protoc 6:743-60.
10. Xia J, Mandal R, Sinelnikov IV, Broadhurst D, Wishart DS. 2012. MetaboAnalyst 2.0--a comprehensive server for metabolomic data analysis. Nucleic Acids Res 40:W127-33.
11. Xia J, Psychogios N, Young N, Wishart DS. 2009. MetaboAnalyst: a web server for metabolomic data analysis and interpretation. Nucleic Acids Res 37:W652-60.
12. Xia J, Sinelnikov IV, Han B, Wishart DS. 2015. MetaboAnalyst 3.0--making metabolomics more meaningful. Nucleic Acids Res 43:W251-7.
13. Storey JD, Tibshirani R. 2003. Statistical significance for genomewide studies. Proc Natl Acad Sci U S A 100:9440-5.
