# Supplementary material for: Phosphoric Metabolites Link Phosphate Import and Polysaccharide Biosynthesis for Candida albicans Cell Wall Maintenance
Source: mBio. 2020 Mar 17;11(2):e03225-19. doi: 10.1128/mBio.03225-19 (PMC7078483; doi:10.1128/mBio.03225-19)
Supplement: TABLE S2 [file mBio.03225-19-st002.pdf]

Table S2. Molecular biologic reagents used in this study.

A. *C. albicans* strains

C. albicans **strain name**	**Parent**	**Genotype**	**Strain background /construction**	**Reference**
SC5314		Wild type		(1)
SN95		*arg4Δ/arg4Δ his1Δ/his1Δ IRO1/iro1Δ::λimm*^{434}<br>*URA3/ura3Δ::λimm*^{434}		(2)
SN152		*arg4Δ/arg4Δ leu2Δ/leu2Δ his1Δ/his1Δ*<br>*URA3/ura3Δ::λimm*^{434}<br>*IRO1/iro1Δ::λimm*^{434}		(2)
JKC2490		bloodstream isolate		This work
JKC915	SC5314	*HIS1/his1:: tetR-FRT*		(3)
JKC917	SN95	*hisΔ1/his1Δ:: tetR-FRT arg4/arg4*<br>*IRO1/iro1Δ::λimm*^{434} *URA3/ura3Δ::λimm*^{434}		(4)
JKC1423	JKC917	*PHO84/pho84::HIS1*<br>*his1/his1:: tetR-FRT arg4/arg4*<br>*IRO1/iro1Δ::λimm*^{434} *URA3/ura3Δ::λimm*^{434}		(4)
JKC1450	JKC1423	*pho84::HIS1/pho84::ARG4*<br>*his1/his1:: tetR-FRT arg4/arg4*<br>*IRO1/iro1Δ::λimm*^{434} *URA3/ura3Δ::λimm*^{434}		(4)
JKC1500	JKC1450	*PHO84-FRT/pho84::ARG4*<br>*his1/his1:: tetR-FRT arg4/arg4*<br>*URA3/ura3::λimm434 IRO1/iro1::λimm434*		(4)
JKC1583	JKC1423	*PHO84/pho84::HIS1*<br>*his1/his1:: tetR-FRT arg4/arg4::ARG4*<br>*IRO1/iro1Δ::λimm*^{434} *URA3/ura3Δ::λimm*^{434}		(4)
JKC1588	JKC1500	*PHO84-FRT/pho84::ARG4 LEU2/leu2::C.d. HIS1*<br>*his1/his1:: tetR-FRT arg4/arg4*<br>*URA3/ura3::λimm*^{434} *IRO1/iro1Δ::λimm*^{434}		(4)
JKC1594	JKC917	*ACT1/* $_{promoter}$*ACT1-NAT1-*$_{terminator}$*ACT1*<br>*his1/his1:: tetR-FRT arg4/arg4*<br>*IRO1/iro1Δ::λimm*^{434} *URA3/ura3Δ::λimm*^{434}		(4)
JKC1596	JKC917	*ACT1/* $_{promoter}$*ACT1-GTR1-NAT1-*$_{terminator}$*ACT1*<br>*his1/his1:: tetR-FRT arg4/arg4*<br>*IRO1/iro1Δ::λimm*^{434} *URA3/ura3Δ::λimm*^{434}		(4)
JKC1598	JKC1450	*ACT1/* $_{promoter}$*ACT1-NAT1-*$_{terminator}$*ACT1*<br>*pho84::HIS1/pho84::ARG4*<br>*his1/his1:: tetR-FRT arg4/arg4*<br>*IRO1/iro1Δ::λimm*^{434} *URA3/ura3Δ::λimm*^{434}		(4)
JKC1600	JKC1450	*ACT1/* $_{promoter}$*ACT1-GTR1-NAT1-*$_{terminator}$*ACT1*<br>*pho84::HIS1/pho84::ARG4*<br>*his1/his1:: tetR-FRT arg4/arg4*<br>*IRO1/iro1Δ::λimm*^{434} *URA3/ura3Δ::λimm*^{434}		(4)
CDH5		*mnn4::hisG-URA3-hisG/* mnn4::hisG		(5)
JKC1659	JKC915	*HIS1/his1Δ:: tetR-FRT*	JKC915 transformed with SnaBI -digested	This work

		PHO84/PHO84 promoter-GFP-NAT1-PHO84	pJK1329, for single-crossover integration of *pPHO84-GFP*	
JKC2111	JKC915	*HIS1/his1::tetR-FRT* *KRE6/kre6::uPAM-FRT*	JKC915 transformed with KpnI/BsiWI digested pJK1428 for *KRE6* heterozygous deletion, after inducing FLP	This work
JKC2113	JKC915	*HIS1/his1::tetR-FRT* *KRE6/kre6::uPAM-FRT*	JKC915 transformed with KpnI/BsiWI digested pJK1428 for *KRE6* heterozygous deletion, after inducing FLP	This work
JKC2174	JKC915	*HIS1/his1::tetR-FRT* *SKN1/skn1::uPAM-FRT*	JKC915 transformed with StuI/BsiWI digested pJK1439 for *SKN1* heterozygous deletion, after inducing FLP	This work
JKC2178	JKC915	*HIS1/his1::tetR-FRT* *SKN1/skn1::uPAM-FRT*	JKC915 transformed with StuI/BsiWI digested pJK1439 for *SKN1* heterozygous deletion, after inducing FLP	This work
JKC2180	JKC2111	*HIS1/his1::tetR-FRT* *KRE6/kre6::uPAM-FRT* *SKN1/skn1::uPAM-FRT*	JKC2111 transformed with StuI/BsiWI digested pJK1439 for *SKN1* heterozygous deletion, after inducing FLP	This work
JKC2184	JKC2111	*HIS1/his1::tetR-FRT* *KRE6/kre6::uPAM-FRT* *SKN1/skn1::uPAM-FRT*	JKC2111 transformed with StuI/BsiWI digested pJK1439 for *SKN1* heterozygous deletion, after inducing FLP	This work
JKC2198	JKC2111	*HIS1/his1::tetR-FRT* *kre6::uPAM-FRT/kre6::FRT-tetO-KRE6*	JKC2111 transformed with KpnI/NcoI digested pJK1447 to place the only allele of *KRE6* under *tetO* control, after inducing FLP	This work
JKC2200	JKC2111	*HIS1/his1::tetR-FRT* *kre6::uPAM-FRT/kre6::FRT-tetO-KRE6*	JKC2111 transformed with KpnI/NcoI digested pJK1447 to place the only allele of *KRE6* under *tetO* control, after inducing FLP	This work
JKC2204	JKC2111	*HIS1/his1::tetR-FRT* *kre6::uPAM-FRT/kre6::FRT-pMal2-KRE6*	JKC2111 transformed with KpnI/NcoI digested pJK1450 to place the only allele of *KRE6* under *pMal2* control, after inducing FLP	This work
JKC2206	JKC2111	*HIS1/his1::tetR-FRT* *kre6::uPAM-FRT/kre6::FRT-pMal2-KRE6*	JKC2111 transformed with KpnI/NcoI digested pJK1450 to place the only allele of *KRE6* under *pMAL2* promoter control, after inducing FLP	This work
JKC2335	JKC2174	*HIS1/his1::tetR-FRT* *skn1::uPAM-FRT/skn1::uPAM-FRT*	JKC2174 transformed with KpnI/BsiWI digested pJK1458 to generate Δ*skn1* homozygous deletion, after inducing FLP	This work
JKC2340	JKC2180	*HIS1/his1::tetR-FRT* *KRE6/kre6::uPAM-FRT* *skn1::uPAM-FRT/skn1::uPAM-FRT*	JKC2180 transformed with KpnI/BsiWI digested pJK1458 to generate Δ*skn1* homozygous deletion, after inducing FLP	This work
JKC2344	JKC2180	*HIS1/his1::tetR-FRT* *KRE6/kre6::uPAM-FRT* *skn1::uPAM-FRT/skn1::uPAM-FRT*	JKC2180 transformed with KpnI/BsiWI digested pJK1458 to generate Δ*skn1* homozygous deletion, after inducing FLP	This work
JKC2350	JKC2200	*HIS1/his1::tetR-FRT* *kre6::uPAM-FRT/kre6::FRT-tetO-KRE6* *SKN1/skn1::uPAM-FRT*	JKC2200 transformed with StuI/BsiWI digested pJK1439 to generate *SKN1* heterozygous deletion, after inducing FLP	This work
JKC2354	JKC2204	*HIS1/his1::tetR-FRT* *kre6::uPAM-FRT/kre6::FRT-pMal2-KRE6* *SKN1/skn1::uPAM-FRT*	JKC2204 transformed with StuI/BsiWI digested pJK1439 to generate *SKN1* heterozygous deletion, after inducing FLP	This work

JKC2389	JKC2344	*HIS1/his1::tetR-FRT kre6::uPAM-FRT/kre6::FRT-pMal2-KRE6 skn1::uPAM-FRT/skn1::uPAM-FRT*	JKC2344 transformed with KpnI/NcoI digested pJK1450 to place the only allele of *KRE6* under *pMAL2* promoter control, after inducing FLP	This work
JKC2391	JKC2344	*HIS1/his1::tetR-FRT kre6::uPAM-FRT/kre6::FRT-pMal2-KRE6 skn1::uPAM-FRT/skn1::uPAM-FRT*	JKC2344 transformed with KpnI/NcoI digested pJK1450 to place the only allele of *KRE6* under *pMAL2* promoter control, after inducing FLP	This work
JKC2395	JKC2344	*HIS1/his1::tetR-FRT kre6::uPAM-FRT/kre6::FRT-tetO-KRE6 skn1::uPAM-FRT/skn1::uPAM-FRT*	JKC2344 transformed with KpnI/NcoI digested pJK1447 to place the only allele of *KRE6* under *tetO* control, after inducing FLP	This work
JKC2530	JKC2113	*HIS1/his1::tetR-FRT kre6::uPAM-FRT/kre6::FRT-tetO-KRE6*	JKC2113 transformed with KpnI/NcoI digested pJK1447 to place the only allele of *KRE6* under *tetO* control, after inducing FLP	This work
JKC2133	JKC1450	*his1/his1::tetR-FRT pho84::ARG4/pho84::HIS1 KRE6/kre6::uPAM-FRT*	JKC1450 transformed with KpnI/BsiWI digested pJK1428 for *KRE6* heterozygous deletion, after inducing FLP	This work
JKC2135	JKC1450	*his1/his1::tetR-FRT pho84::ARG4/pho84::HIS1 KRE6/kre6::uPAM-FRT*	JKC1450 transformed with KpnI/BsiWI digested pJK1428 for *KRE6* heterozygous deletion, after inducing FLP	This work
JKC2294	JKC1450	*his1/his1::tetR-FRT pho84::ARG4/pho84::HIS1 SKN1/skn1::uPAM-FRT*	JKC1450 transformed with StuI/BsiWI digested pJK1439 for *SKN1* heterozygous deletion, after inducing FLP	This work
JKC2296	JKC1450	*his1/his1::tetR-FRT pho84::ARG4/pho84::HIS1 SKN1/skn1::uPAM-FRT*	JKC1450 transformed with StuI/BsiWI digested pJK1439 for *SKN1* heterozygous deletion, after inducing FLP	This work
JKC2302	JKC2133	*his1/his1::tetR-FRT pho84::ARG4/pho84::HIS1 KRE6/kre6::uPAM-FRT SKN1/skn1::uPAM-FRT*	JKC2133 transformed with StuI/BsiWI digested pJK1439 for *SKN1* heterozygous deletion, after inducing FLP	This work
JKC2304	JKC2133	*his1/his1::tetR-FRT pho84::ARG4/pho84::HIS1 KRE6/kre6::uPAM-FRT SKN1/skn1::uPAM-FRT*	JKC2133 transformed with StuI/BsiWI digested pJK1439 for *SKN1* heterozygous deletion, after inducing FLP	This work
JKC2464	JKC2135	*his1/his1::tetR-FRT pho84::ARG4/pho84::HIS1 kre6::uPAM-FRT/kre6::FRT-pMal2-KRE6*	JKC2135 transformed with KpnI/NcoI digested pJK1450 to place the only allele of *KRE6* under *pMAL2* promoter control, after inducing FLP	This work
JKC2474	JKC2133	*his1/his1::tetR-FRT pho84::ARG4/pho84::HIS1 kre6::uPAM-FRT/kre6::FRT-pMal2-KRE6*	JKC2133 transformed with KpnI/NcoI digested pJK1450 to place the only allele of *KRE6* under *pMAL2* promoter control, after inducing FLP	This work
JKC2478	JKC2135	*his1/his1::tetR-FRT pho84::ARG4/pho84::HIS1 kre6::uPAM-FRT/kre6::FRT-tetO-KRE6*	JKC2135 transformed with KpnI/NcoI digested pJK1447 to place the only allele of *KRE6* under *tetO* control, after inducing FLP	This work
JKC2378	JKC2302	*his1/his1::tetR-FRT pho84::ARG4/pho84::HIS1 KRE6/kre6::uPAM-FRT skn1::uPAM-FRT/skn1::uPAM-FRT*	JKC2302 transformed with KpnI/BsiWI digested pJK1458 to generate Δ*skn1* homozygous deletion, after inducing FLP	This work
JKC2468	JKC2378	*his1/his1::tetR-FRT pho84::ARG4/pho84::HIS1 skn1::uPAM-FRT/skn1::uPAM-FRT kre6::uPAM-FRT/kre6::FRT-pMal2-KRE6*	JKC2378 transformed with KpnI/NcoI digested pJK1450 to place the only allele of *KRE6* under *pMAL2* promoter control, after inducing FLP	This work

JKC2125	JKC915	*HIS1/his1::tetR-FRT*<br>*CHS1/chs1::uPAM-FRT*	JKC915 transformed with KpnI/BsiWI digested pJK1431 for *CHS1* heterozygous deletion, after inducing FLP	This work
JKC2128	JKC915	*HIS1/his1::tetR-FRT*<br>*CHS1/chs1::uPAM-FRT*	JKC915 transformed with KpnI/BsiWI digested pJK1431 for *CHS1* heterozygous deletion, after inducing FLP	This work
JKC2212	JKC2128	*HIS1/his1::tetR-FRT*<br>*chs1::uPAM-FRT/chs1::FRT-tetO-CHS1*	JKC2128 transformed with KpnI/NcoI digested pJK1449 to place the only allele of *CHS1* under *tetO* control, after inducing FLP	This work
JKC2214	JKC2128	*HIS1/his1::tetR-FRT*<br>*chs1::uPAM-FRT/chs1::FRT-tetO-CHS1*	JKC2128 transformed with KpnI/NcoI digested pJK1449 to place the only allele of *CHS1* under *tetO* control, after inducing FLP	This work
JKC2216	JKC2128	*HIS1/his1::tetR-FRT*<br>*chs1::uPAM-FRT/chs1::FRT-pMal2-CHS1*	JKC2128 transformed with KpnI/NcoI digested pJK1452 to place the only allele of *CHS1* under *pMAL2* promoter control, after inducing FLP	This work
JKC2218	JKC2128	*HIS1/his1::tetR-FRT*<br>*chs1::uPAM-FRT/chs1::FRT-pMal2-CHS1*	JKC2128 transformed with KpnI/NcoI digested pJK1452 to place the only allele of *CHS1* under *pMAL2* promoter control, after inducing FLP	This work
JKC2272	JKC2125	*HIS1/his1::tetR-FRT*<br>*chs1::uPAM-FRT/chs1::FRT-tetO-CHS1*	JKC2125 transformed with KpnI/NcoI digested pJK1449 to place the only allele of *CHS1* under *tetO* control	This work
JKC2274	JKC2125	*HIS1/his1::tetR-FRT*<br>*chs1::uPAM-FRT/chs1::FRT-tetO-CHS1*	JKC2125 transformed with KpnI/NcoI digested pJK1449 to place the only allele of *CHS1* under *tetO* control, after inducing FLP	This work
JKC2280	JKC2125	*HIS1/his1::tetR-FRT*<br>*chs1::uPAM-FRT/chs1::FRT-pMal2-CHS1*	JKC2125 transformed with KpnI/NcoI digested pJK1452 to place the only allele of *CHS1* under *pMAL2* promoter control, after inducing FLP	This work
JKC2137	JKC1450	*his1/his1::tetR-FRT*<br>*pho84::ARG4/pho84::HIS1*<br>*CHS1/chs1::uPAM-FRT*	JKC1450 transformed with KpnI/BsiWI digested pJK1431 for *CHS1* heterozygous deletion, after inducing FLP	This work
JKC2141	JKC1450	*his1/his1::tetR-FRT*<br>*pho84::ARG4/pho84::HIS1*<br>*CHS1/chs1::uPAM-FRT*	JKC1450 transformed with KpnI/BsiWI digested pJK1431 for *CHS1* heterozygous deletion, after inducing FLP	This work
JKC2234	JKC2141	*his1/his1::tetR-FRT*<br>*pho84::ARG4/pho84::HIS1*<br>*chs1::uPAM-FRT/chs1::FRT-pMal2-CHS1*	JKC2141 transformed with KpnI/NcoI digested pJK1452 to place the only allele of *CHS1* under *pMAL2* promoter control, after inducing FLP	This work
JKC2288	JKC2141	*his1/his1::tetR-FRT*<br>*pho84::ARG4/pho84::HIS1*<br>*chs1::uPAM-FRT/chs1::FRT-tetO-CHS1*	JKC2141 transformed with KpnI/NcoI digested pJK1449 to have the only allele of *CHS1* under *tetO* promoter, after inducing FLP	This work
JKC2290	JKC2141	*his1/his1::tetR-FRT*<br>*pho84::ARG4/pho84::HIS1*<br>*chs1::uPAM-FRT/chs1::FRT-tetO-CHS1*	JKC2141 transformed with KpnI/NcoI digested pJK1449 to place the only allele of *CHS1* under *tetO* control, after inducing FLP	This work
JKC2319	JKC2137	*his1/his1::tetR-FRT*<br>*pho84::ARG4/pho84::HIS1*<br>*chs1::uPAM-FRT/chs1::FRT-tetO-CHS1*	JKC2137 transformed with KpnI/NcoI digested pJK1449 to place the only allele of *CHS1* under *tetO* control, after inducing FLP	This work

B. Plasmids

Plasmid	Description	Source (Reference)
pJK1000	*FLP-NAT1 tetO-PES1* construct, vector backbone is pLitmus28 (New England Biolabs)	(3)
pJK1329	*pPHO84-GFP* construct for single-crossover integration. Product of fjk1625 and rjk1670 using SC5314 genomic DNA as template, ligated into a GFP-expressing (amplified from pGFP-HIS1 (6) with fjk1615 and rjk1633) derivative of pJK1027 (7) using KpnI and ClaI sites.	This work
pJK1351	*FLP-NAT1 tetO-SOD3* construct, derived from pJK1000. Product of fjk1821 and r1822 using SC5314 genomic DNA as template and product of fjk1819 and r1820 using SC5314 genomic DNA as template were ligated into pJK1000 using SacII/NcoI and KpnI/ApaI sites, respectively.	(8)
pJK1353	*FLP-NAT1 pMAL2-SOD3* construct, derived from pJK1351. pMAL2 promoter was ligated into p1351 using NotI and SacII sites.	(8)
pJK1364	*FLP-NAT1 pho87 deletion* intermediate construct, derived from pJK1351. Product of fjk1848 and r1849 using SC5314 genomic DNA as template was ligated into pJK1351 using NotI/NcoI sites.	This work
pJK1372	*FLP-NAT1 pho87 deletion* construct, derived from pJK1364. Product of fjk1846 and r1862 using SC5314 genomic DNA as template was ligated into pJK1364 using KpnI/ApaI sites.	This work
pJK1428	*FLP-NAT1 kre6 deletion* construct, derived from pJK1372. Product of fjk1974 and r1975 using SC5314 genomic DNA as template and product of fjk1976 and r1977 using SC5314 genomic DNA as template were ligated into pJK1372 using KpnI/AscI and NotI/BsiWI sites, respectively.	This work
pJK1439	*FLP-NAT1 skn1-1st allele deletion* construct, derived from pJK1372. Product of fjk1982 and r1983 using SC5314 genomic DNA as template and product of fjk1984 and r1985 using SC5314 genomic DNA as template were ligated into pJK1372 using KpnI/AscI and NotI/BsiWI sites, respectively.	This work
pJK1447	*FLP-NAT1 tetO-KRE6* construct, derived from pJK1351. Product of fjk1990 and r1991 using SC5314 genomic DNA as template and product of fjk1992 and r1993 using SC5314 genomic DNA as template were ligated into pJK1351 using KpnI/ApaI and SacII/NcoI sites, respectively.	This work
pJK1450	*FLP-NAT1 pMAL2-KRE6* construct, derived from pJK1353. Product of fjk1990 and r1991 using SC5314 genomic DNA as template and product of fjk1992 and r1993 using SC5314 genomic DNA as template were ligated into pJK1351 using KpnI/ApaI and SacII/NcoI sites, respectively.	This work
pJK1458	*FLP-NAT1 skn1-2nd allele deletion* construct, derived from pJK1372. Product of fjk2009 and r2010 using SC5314 genomic DNA as template and product of fjk2011 and r2012 using SC5314 genomic DNA as template were ligated into pJK1372 using KpnI/AscI and NotI/BsiWI sites, respectively.	This work
pJK1431	*FLP-NAT1 chs1 deletion* construct, derived from pJK1372. Product of fjk1968 and r1969 using SC5314 genomic DNA as template and product of fjk1970 and r1971 using SC5314 genomic DNA as template were ligated into pJK1372 using KpnI/AscI and NotI/BsiWI sites, respectively.	This work
pJK1449	*FLP-NAT1 tetO-CHS1* construct, derived from pJK1351. Product of fjk1996 and r1997 using SC5314 genomic DNA as template and product of fjk1998 and r1999 using SC5314 genomic DNA as template were ligated into pJK1351 using KpnI/ApaI and SacII/NcoI sites, respectively.	This work
pJK1452	*FLP-NAT1 pMAL2-CHS1* construct, derived from pJK1353. Product of fjk1996 and r1997 using SC5314 genomic DNA as template and product of fjk1998 and r1999 using SC5314 genomic DNA as template were ligated into pJK1353 using KpnI/ApaI and SacII/NcoI sites, respectively.	This work

C. Oligonucleotides

Primer name	Purpose	Sequence 5' to 3'<br>(lower cases - restriction enzyme recognition sites)
fjk1615	Forward primer to amplify GFP	CCTGCTatcgatATGTCTAAAGGTGAAGAATTAT
rjk1633	Reverse primer to amplify GFP	GCAGCTcccgggTTATTTGTATAATTCATCCATACCATGG
fjk1625	Forward primer to amplify *PHO84* promoter	GCACAGggtaccTTGAGAGGATTTGGTGGAATAGGT
rjk1670	Reverse primer to amplify *PHO84* promoter	GTATCTGTAgacgtcTAGTGTGAGTTGAATTTCAGCAA
fjk1685	Forward primer to verify 5' end of integration of pJK1329, first primer set of nested PCR	ATTGTCGTGCAAATGGTTGA
rjk1692	Reverse primer to verify 5' end of integration of pJK1329, first primer set of nested PCR	CAACCAAAATTGGGACAACA
fjk1686	Forward primer to verify 5' end of integration of pJK1329, second primer set of nested PCR	CGTGCAAATGGTTGAGCTAC
rjk1515	Reverse primer to verify 5' end of integration of pJK1329, second primer set of nested PCR	CCAGTAAATAATTCTTCACC
fjk1517	Forward primer to verify 3' end of integration of pJK1329,	GGAATTGTGAGCGGATAAC
rjk1365	Reverse primer to verify 3' end of integration of pJK1329,	GCAGCCATAATAGCACCTCT
fjk490	Forward primer to verify the 3'end integration of '*FLP-NAT1'* cassette containing constructs	TCAAGGAGGGTATTCTGGGC
rjk1339	Reverse primer to verify the 5'end of integration of *'FLP-NAT1'* cassette containing constructs	TGGTGTGTTGTTGACAGGCAAC
fjk1974	Forward primer to amplify the *kre6* deletion upstream homologous sequence	CATCAGggtaccTCCCATTACATTCGTTTCAACA
rjk1975	Reverse primer to amplify the *kre6* deletion upstream homologous sequence	GATggcgcgccGAAGTTAAATCTCTTTGAGACGCC
fjk1976	Forward primer to amplify the *kre6* deletion downstream homologous sequence	AAGGTAAGCAgcggccgcAACTTCATTTGAAGATGGTGGA
rjk1977	Reverse primer to amplify the *kre6* deletion downstream homologous sequence	CTCATGcgtacgTCTTCAATAACTAGCCAATACATACACA
fjk1978	Forward primer to verify the 5'end of *kre6* deletion mutant	GTTTTTCCCTTTCCCCTTCA
rjk1979	Reverse primer to verify the 3'end of *kre6* deletion mutant	TTTGTTTGATAGACGGGATGG
fjk1982	Forward primer to amplify the *skn1* deletion-1st allele upstream homologous sequence	CATCAGggtaccCACCACCAATGCCAATGTAA
rjk1983	Reverse primer to amplify the *skn1* deletion-1st allele upstream homologous sequence	GATggcgcgccTGTAAGTTGTATTAAAAGTTTAAGTTTTTAG TAGC

fjk1984	Forward primer to amplify the *skn1* deletion-1st allele downstream homologous sequence	AAGGTAAGCAgcggccgcAACACCAACCCCCCTATATTTAC
rjk1985	Reverse primer to amplify the *skn1* deletion-1st allele downstream homologous sequence	CTCATGcgtacgCCAAAATGGAATCAAAACGA
fjk1986	Forward primer to verify the 5'end of *skn1* deletion mutant	GTGAATAAGTGGTGTGGGAATG
rjk1987	Reverse primer to verify the 3'end of *skn1* deletion mutant	AAAGACTCCACCACCACCAG
fjk1990	Forward primer to amplify the *tetO/pMAL2-KRE6* upstream homologous sequence	CATCCGggtaccCCTTTCCCCTTCAACAACAA
rjk1991	Reverse primer to amplify the *tetO/pMAL2-KRE6* upstream homologous sequence	GATCgggcccTCGAAAGAAATTACAAGAAAAACAA
fjk1992	Forward primer to amplify the *tetO/pMAL2-KRE6* downstream homologous sequence	GGATCCccgcggATGGCGTCTCAAAGAGATTTAACTTC
rjk1993	Reverse primer to amplify the *tetO/pMAL2-KRE6* downstream homologous sequence	CTCATGccatggCAGTGAACTACCACCATTTGAA
fjk1994	Forward primer to verify the 5'end of *tetO/pMAL2-KRE6* integration	AGACGCCGAAAGTGAAAGAA
rjk1995	Reverse primer to verify the 3'end of *tetO/pMAL2-KRE6* integration	AATTCTGCTGGACTGGTTGG
fjk2009	Forward primer to amplify the *skn1* deletion-2nd allele upstream homologous sequence	CATCAGggtaccATGGAAAGAGATTTGACTTATAATGC
rjk2010	Reverse primer to amplify the *skn1* deletion-2nd allele upstream homologous sequence	GCTggcgcgccCATGTTGTTGTGATGGAATACG
fjk2011	Forward primer to amplify the *skn1* deletion-2nd allele downstream homologous sequence	AAGGTAAGCTgcggccgcTGGTAATGTTGGTTGGAGAAA
rjk2012	Reverse primer to amplify the *skn1* deletion-2nd allele downstream homologous sequence	CTCATGcgtacgTTAACATCCAATTAATTTATGTTTGG
fjk1968	Forward primer to amplify the *chs1* deletion upstream homologous sequence	CATCAGggtaccGCTTCAAGGGAAAAGGTGGT
rjk1969	Reverse primer to amplify the *chs1* deletion upstream homologous sequence	GATggcgcgccGATATTATGTAGTTTAAAGGGTATTCTGG
fjk1970	Forward primer to amplify the *chs1* deletion downstream homologous sequence	AAGGTAAGCAgcggccgcCCCTCCCTCTAAAATGAAGACC
rjk1971	Reverse primer to amplify the *chs1* deletion downstream homologous sequence	CTCATGcgtacgGACTTGGCCTTTGCGTATTC
fjk1972	Forward primer to verify the 5'end of *chs1* deletion mutant	GGGGGAACATTTACTCAGCTC
rjk1973	Reverse primer to verify the 3'end of *chs1* deletion mutant	AAGTGTGCATACCACCGACA
fjk1996	Forward primer to amplify the *tetO/pMAL2-CHS1* upstream homologous sequence	CATCCAggtaccGCTTTTGGCTCTTGTTTGGA
rjk1997	Reverse primer to amplify the *tetO/pMAL2-CHS1* upstream homologous sequence	GATCgggcccCGCAGATCCATTTTCAAATTC
fjk1998	Forward primer to amplify the *tetO/pMAL2-CHS1* downstream homologous sequence	GGATCCccgcggATGAAGAATCCATTTGACAGTGG
rjk1999	Reverse primer to amplify the *tetO/pMAL2-CHS1* downstream homologous sequence	CTCATGccatggGAATTGTGCTCTTGGTGTGG
fjk2000	Forward primer to verify the 5'end of *tetO/pMAL2-CHS1* integration	TCGTTGCAACAAGCAAAAGT
rjk2001	Reverse primer to verify the 3'end of *tetO/pMAL2-CHS1* integration	TTGTGGAGGAGGCAAAAATC

D. Antibodies

Purpose	Antigen recognized	Species	Source or Reference
primary	Mkc1	rabbit	Gift from Dr. Jesus Pla
primary	P-Mkc1	rabbit	Cell Signaling Technology, cat. #4370P
loading control	PSTAIRE	rabbit	Santa Cruz Biotechnology, cat. # sc-53
dot blot and ELISA	beta-1,6-glucan (pustulan)	rabbit	(9)
secondary	Rabbit Ig	goat	Cell Signaling Technology, cat. # 7074s

References for Molecular biologic reagents

1. Fonzi WA, Irwin MY. 1993. Isogenic strain construction and gene mapping in Candida albicans. Genetics 134:717-28.
2. Noble SM, Johnson AD. 2005. Strains and strategies for large-scale gene deletion studies of the diploid human fungal pathogen Candida albicans. Eukaryot Cell 4:298-309.
3. Shen J, Cowen LE, Griffin AM, Chan L, Köhler JR. 2008. The Candida albicans pescadillo homolog is required for normal hypha-to-yeast morphogenesis and yeast proliferation. Proc Natl Acad Sci U S A 105:20918-23.
4. Liu NN, Flanagan PR, Zeng J, Jani NM, Cardenas ME, Moran GP, Köhler JR. 2017. Phosphate is the third nutrient monitored by TOR in Candida albicans and provides a target for fungal-specific indirect TOR inhibition. Proc Natl Acad Sci U S A doi:10.1073/pnas.1617799114.
5. Hobson RP, Munro CA, Bates S, MacCallum DM, Cutler JE, Heinsbroek SE, Brown GD, Odds FC, Gow NA. 2004. Loss of cell wall mannosylphosphate in Candida albicans does not influence macrophage recognition. J Biol Chem 279:39628-35.
6. Gerami-Nejad M, Berman J, Gale CA. 2001. Cassettes for PCR-mediated construction of green, yellow, and cyan fluorescent protein fusions in Candida albicans. Yeast 18:859-64.
7. Patenaude C, Zhang Y, Cormack B, Köhler J, Rao R. 2013. Essential role for vacuolar acidification in Candida albicans virulence. J Biol Chem 288:26256-64.
8. Liu NN, Uppuluri P, Broggi A, Besold A, Ryman K, Kambara H, Solis N, Lorenz V, Qi W, Acosta Zaldivar M, Emami SN, Bao B, An D, Bonilla F, Sola-Visner M, Filler S, Luo HR, Engstrom Y, Ljungdahl PO, Culotta VC, Zanoni I, Lopez-Ribot JL, Köhler JR. 2018. Intersection of phosphate transport, oxidative stress and TOR signalling in Candida albicans virulence. PLoS Pathogens 14:e1007076.
9. Kottom TJ, Hebrink DM, Jenson PE, Gudmundsson G, Limper AH. 2015. Evidence for Proinflammatory beta-1,6 Glucans in the Pneumocystis carinii Cell Wall. Infect Immun 83:2816-26.
